# Determination of Gene‐Level Fitness Contributions Across the Mucin‐Utilization Network of *Bacteroides thetaiotaomicro* *n*

**DOI:** 10.1002/advs.78118

**Published:** 2026-10-04

**Authors:** Kangsan Kim, Jiwon Lee, Minjeong Kang, Sung Sun Yim, Donghui Choe, Byung‐Kwan Cho

**Affiliations:** ^1^ Graduate School of Engineering Biology Korea Advanced Institute of Science and Technology Daejeon Republic of Korea; ^2^ KAIST Institute for the Bioinnovation Korea Advanced Institute of Science and Technology Daejeon Republic of Korea; ^3^ Department of Biological Sciences Korea Advanced Institute of Science and Technology Daejeon Republic of Korea; ^4^ Department of Biological Sciences Sungkyunkwan University Suwon Republic of Korea

**Keywords:** bacteroides thetaiotaomicron, CRISPRi, gene fitness, mucin, systems biology

## Abstract

Host mucus is a major nutrient reservoir for gut *Bacteroidota*, yet the gene‐level determinants of mucin utilization remain poorly defined. In this study, we used a genome‐scale CRISPR interference library in *Bacteroides thetaiotaomicron* to systematically map fitness contributions across the mucin‐utilization network, spanning surface accessibility, glycan degradation, and intracellular sugar assimilation. Our screen identified 1,256 genes with condition‐dependent fitness divergence, which revealed a layered organization of mucin‐dependent growth. A prominent mucin‐specific fitness signature was enrichment of strains with repressed capsular polysaccharide loci. Subsequent competition and deletion experiments validated that specific capsules impede growth on mucin, supporting a surface‐accessibility trade‐off model. By contrast, most mucin‐induced carbohydrate‐binding and glycoside hydrolase genes showed negligible fitness contributions, consistent with functional redundancy in extracellular glycan capture and processing. Conversely, downstream sugar catabolism showed pronounced condition‐specific constraints, with the galactose‐utilization gene BT_0623, causing measurable fitness defects upon repression in both mucin‐ and galactose‐containing media. Finally, comparison with murine colon CRISPRi data revealed broad concordance with the in vitro mucin fitness landscape, while also highlighting environment‐specific differences, in CPS‐ and polysaccharide degradation‐associated functions. Together, these results define mucin utilization as a layered fitness architecture governed by surface‐accessibility costs, redundant extracellular glycan processing, and downstream metabolic constraints.

## Graphical abstract

Genome‐wide CRISPRi and RNA‐seq resolve how *B. thetaiotaomicron* converts host mucosal glycans into fitness. The study reveals a layered mucin‐utilization architecture in which capsular polysaccharides constrain surface access, extracellular CAZymes and peptidases show functional redundancy, and intracellular catabolic pathways create discrete metabolic bottlenecks, linking in vitro glycan foraging to host colonization.

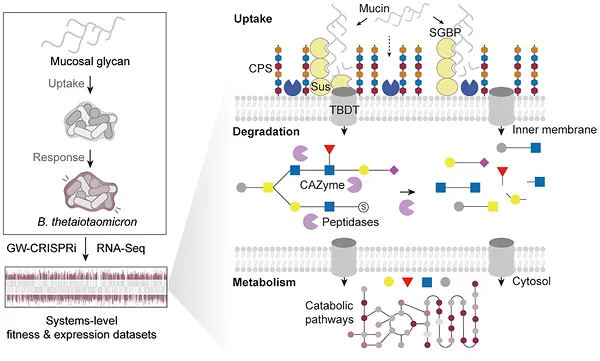

## 1 Introduction

The human gut symbiont *Bacteroides thetaiotaomicron* is a prominent model for understanding microbiome physiology and ecological interactions within the mammalian gut [1]. A defining feature of the intestinal niche is the mucus layer, where heavily glycosylated mucins provide a host‐derived carbon reservoir that can sustain microbial growth when dietary polysaccharides are limited [2, 3]. Consistent with this niche, *B. thetaiotaomicron* encodes an extensive repertoire of Carbohydrate Active Enzymes (CAZymes) and Polysaccharide Utilization Loci (PULs) that enable the degradation and assimilation of host‐derived glycans [4, 5, 6]. However, the utilization of complex mucin glycans is a multilayered metabolic network involving substrate access at the cell surface, glycan degradation, and intracellular assimilation of released sugars [5, 7, 8]. Therefore, defining the specific roles and essentiality of individual genes is critical for understanding how this bacterium adapts to dynamic nutritional shifts. Despite this complexity, the individual fitness contributions of genes within this broader mucin‐utilization network remain incompletely defined.

In *B. thetaiotaomicron*, transcriptomic and functional studies have identified extensive repertoires of carbohydrate‐binding proteins, glycoside hydrolases, and downstream catabolic enzymes associated with these stages of mucin processing [4, 6, 7, 9]. Previous studies have provided important insights into host colonization and in vivo fitness. For example, transcriptome analysis and in vivo transposon screens have demonstrated that the fitness contributions of specific PULs and CAZymes are highly dynamic and heavily dependent on the host's specific dietary features [3, 10, 11, 12]. It was also revealed that, during nutrient limitation, the capacity to exploit host‐secreted mucus provides an important adaptive advantage for persistence within the gut [13]. While these results established the global importance of mucin utilization for persistence, they have not systematically resolved individual gene functions under mucin‐defined growth conditions. Therefore, key fitness determinants within the mucin utilization network remain unresolved. A gene‐by‐gene fitness map is therefore necessary to refine our understanding of this biological process at gene‐level resolution.

CRISPRi is well suited to address these questions as it enables pooled, genome‐scale interrogation of gene function while retaining access to both essential and non‐essential processes [14]. Here we constructed a genome‐scale CRISPRi library in *B. thetaiotaomicron* and used it to compare fitness landscapes in glucose‐ and mucin‐based minimal media. By integrating with transcriptomic profiling, targeted genetic validation, and parallel pooled measurements during murine colonization, this framework allowed us to map the gene‐level fitness architecture of mucin utilization in *Bacteroides thetaiotaomicron*, identifying the genetic constraints that define growth on host‐derived mucin.

## 2 Results

### 2.1 The Mucin Utilization Network Model in *B. thetaiotaomicron*

Guided by the current model of mucin utilization in *B. thetaiotaomicron*, we reconstructed this process as a directional nutrient‐acquisition network spanning substrate encounter at the cell surface, mucin *O*‐glycan deconstruction and transport, and downstream assimilation of released sugars into central metabolism (Figure 1). At the outer membrane, surface glycan binding proteins (SGBP), including SusD‐like and accessory surface glycan‐binding proteins, TonB‐dependent transporters (TBDT), and surface‐localized CAZymes with carbohydrate‐binding modules (CBMs) promote glycan recognition, capture, and retention. These surface‐associated CAZymes also contribute to glycan degradation, and TBDTs mediate oligosaccharide uptake [15, 16, 17, 18]. In parallel, capsular polysaccharides may indirectly influence mucin access by altering the steric properties of the cell surface [17] (Figure 1a). Although direct evidence for CPS‐mediated control of mucin access remains limited, the coordinated regulation of host‐glycan‐responsive PULs and capsule biosynthesis suggests that capsule remodeling is integrated with glycan‐acquisition programs [19]. Following substrate encounter, mucin processing proceeds through coordinated activation of PULs, which couple glycan sensing and TonB‐dependent transport with the activities of CAZymes (Figure 1b), in particular, glycoside hydrolases, sulfatases and mucin‐associated peptidases that degrade mucin *O*‐glycans into smaller oligo‐, mono‐saccharides and glycopeptides [5, 20, 21, 22]. The resulting complex oligosaccharides are then captured and imported into the periplasm via TBDTs, where a consortium of CAZymes further depolymerize them into transportable mono‐ and disaccharides. *Bacteroides* utilizes mucin‐derived sugars liberated from mucin, where major constituents include fucose, galactose, *N*‐acetylglucosamine (GlcNAc) and *N*‐acetylgalactosamine (GalNAc) [23]. These liberated monosaccharides are routed through highly specific, dedicated catabolic pathways [7, 18], and *B. thetaiotaomicron* is predicted to encode each of this catabolic pathway (Figure 1c). For instance, it encodes a fucose utilization operon that enables isomerization and phosphorylation of fucose before entering central metabolism [2, 24]. In addition, it also encodes the Leloir pathway, as well as genes associated with transport of hexosamines into glycolysis [25]. As the result, it is expected that *B. thetaiotaomicron* utilize these catabolic pathways to funnel carbon into the central metabolic pathways.

**FIGURE 1:**
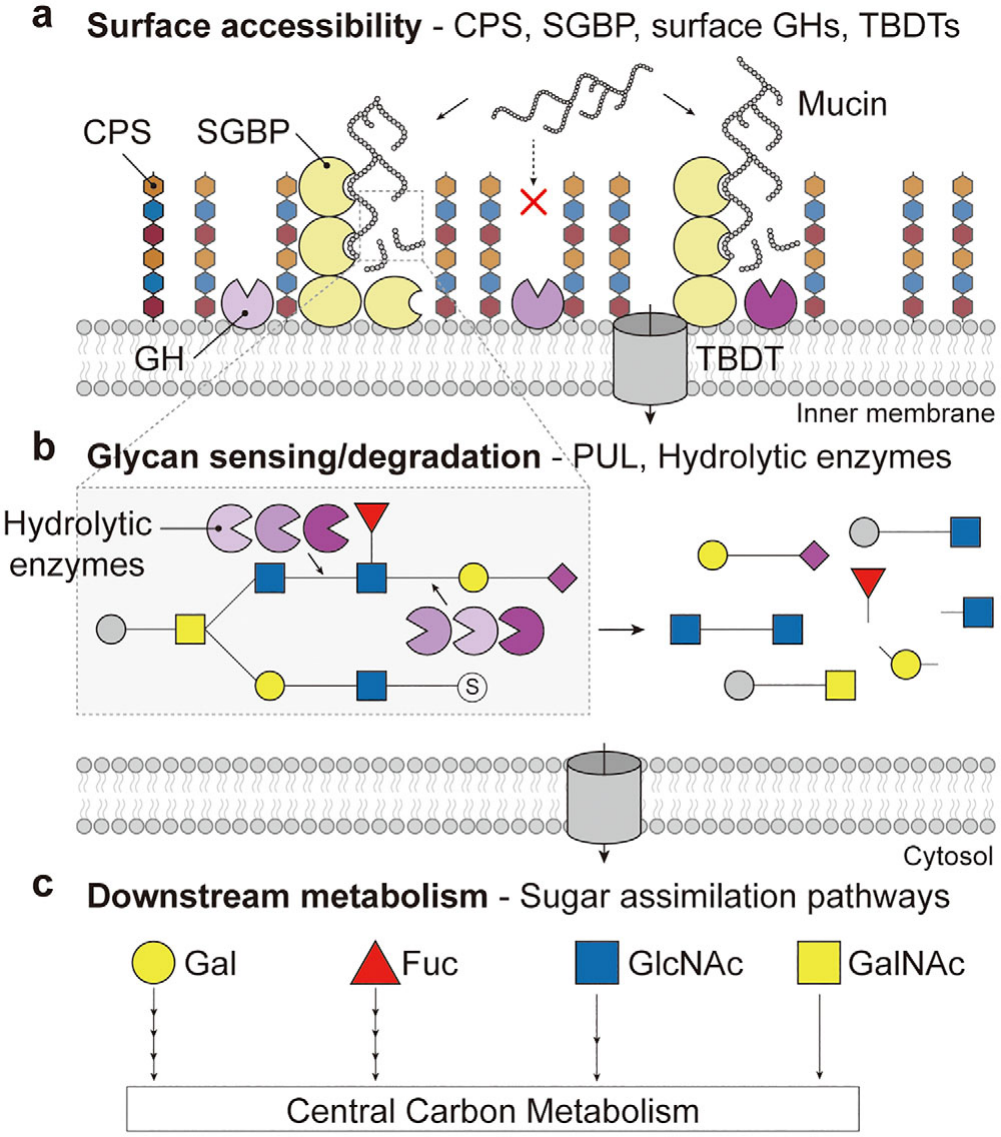
A simplified schematic representation of the mucin utilization network of *B. thetaiotaomicron* visualized in a three‐tiered model. (a) Surface accessibility—At the outer membrane, CPS layers form a physical barrier that may restrict the access of bulky extracellular mucin to surface‐embedded SGBPs for membrane localization, and for downstream mucin degradation by surface hydrolytic enzymes. (b) Glycan degradation—Complex branched oligosaccharides are then sensed and hydrolyzed by a catalog of membrane bound proteins and hydrolytic enzymes into simpler oligosaccharides and constituent sugars. (c) Downstream metabolism—Liberated mucin constituent sugars including galactose, fucose, GlcNAc, and GalNAc are assimilated into central carbon metabolism via dedicated catabolic networks.

While each of these functional ‘modules’ has been characterized individually based on transcriptomic and functional genetic studies, their relative contributions to mucin‐dependent growth and the extent to which transcriptional induction predicts functional importance have remained unresolved at genome scale. To systematically interrogate the fitness contribution of genetic constituents associated in each layer of this network, we combined genome‐wide CRISPRi fitness profiling with RNA‐Seq during growth on mucin as the sole carbon source.

### 2.2 Construction of a Genome‐Wide sgRNA Library

To enable the high‐throughput functional characterization of the entire *B. thetaiotaomicron* genome, we used a plasmid‐based inducible CRISPRi system previously implemented in this strain (Figure S1a, b) [26]. The system utilizes a catalytically inactive Cas9 (dCas9) integrated into a neutral locus of the *B. thetaiotaomicron* chromosome, combined with an optimized, highly expressed sgRNA cassette driven by the strong synthetic P_BfP1E6 promoter.

While CRISPRi is a powerful tool for transcriptional regulation, its efficacy is frequently affected by genomic context and the stochastic nature of dCas9‐DNA binding [27, 28]. A single optimized site may not yield reproducible repression across varying physiological or environmental states. Therefore, we designed a high‐resolution, genome‐wide CRISPRi library comprising 55,378 distinct sgRNAs targeting the non‐template strands of all coding sequences (CDS), with target sites spaced approximately 100 bp (Figure 2a, Table S1). This dense mapping strategy circumvents the inconsistencies of genomic influence by providing comprehensive coverage across the entire transcriptional landscape [29].

**FIGURE 2:**
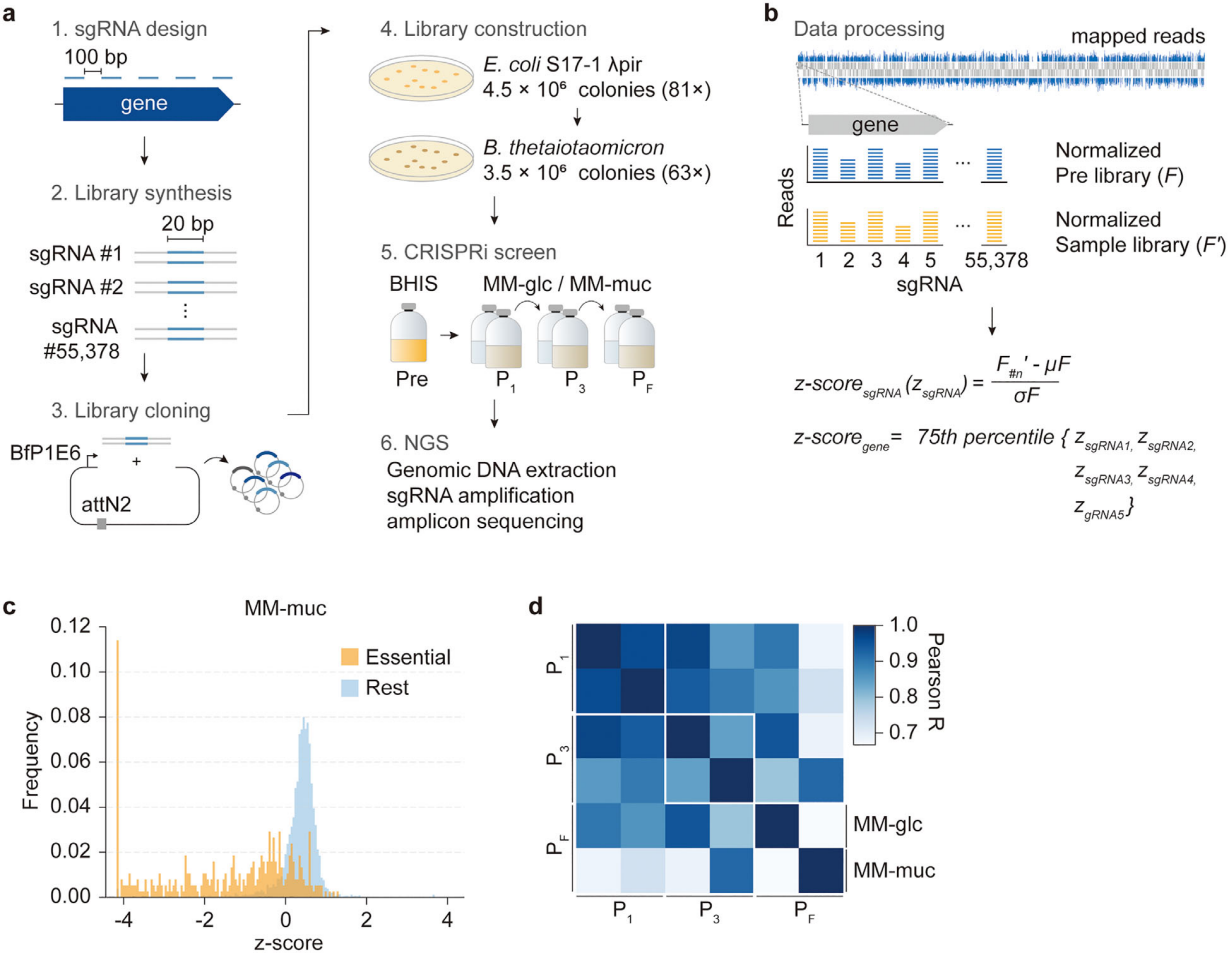
Construction of a genome‐scale CRISPRi screening platform in *Bacteroides thetaiotaomicron*. (a) Schematic overview of the CRISPRi screening workflow. A highly saturated sgRNA library was computationally designed to target CDS at roughly 100‐bp intervals. The library was transformed into the donor *Escherichia coli* S17‐1 λpir and conjugated into *B. thetaiotaomicron*. The library populations were subjected to selection in MM‐muc or glucose MM‐glc across passages. After sampling, the sgRNA libraries were sequenced using next‐generation sequencing (NGS). (b) z‐score of a specific sgRNA (z‐score_sgRNA_) was calculated as (*F_#n_′—µF*) / *σF*, where *F_#n_′* denotes the normalized individual sgRNA counts in the sample library, and *µF* and *σF* denote the mean and standard deviation of the Pre library, respectively. The gene‐level z‐score (z‐score_gene_) is determined by the 75th percentile of the constituent sgRNA z‐scores. (c) Distribution of z‐scores for sgRNAs targeting established core essential genes compared to the rest of the genome following growth in MM‐muc. (d) Heatmap detailing the Pearson correlation coefficients of genome‐wide fitness profiles across the sequential passages in MM‐glc and MM‐muc conditions.

The synthesized sgRNA library was introduced into the conjugative donor *E. coli* S17‐1 λpir, yielding 4.5 × 10^6^ colonies, which represents 81‐fold coverage of the sgRNA pool. The library was conjugated into the dCas9‐expressing *B. thetaiotaomicron*, resulting in approximately 3.5 × 10^6^ colonies equivalent to 63‐fold coverage of the designed sgRNAs (refer to Figure S1c–f and Method for detailed procedures of CRISPRi library construction). Sequencing of the initial library (pre) confirmed that 53,768 individual sgRNAs were retained (97.1% of the initial sgRNA pool) with a median read count of 128.2, providing sufficient depth for downstream analysis (Figure S2a, Tables S2 and S3). In addition, we observed a strong linear correlation (Pearson R = 0.98) between gene length and the number of unique sgRNAs per gene, confirming that the designed sgRNA pool was well retained through the conjugation process (Figure S2b). Finally, changes in sgRNA abundance relative to their positions within genes showed no statistical differences (Figure S2c, d), suggesting an unbiased inhibitory activity of the dCas9‐sgRNA complex [30].

### 2.3 Genome‐Wide Gene Fitness Analysis Based on CRISPRi Screening

The initial CRISPRi library was pre‐cultured in BHIS liquid medium and subsequently propagated in minimal media supplemented with either mucin (MM‐muc) or glucose (MM‐glc) for six and seven passages, respectively, encompassing 4–5 generations per passage for a total of approximately 30 generations. We analyzed at the first (P_1_), third (P_3_), and final (P_F_) passages (Figure 2a) and calculated z‐scores to determine relative enrichment or depletion of the individual genes. The 75th percentile of all z‐scores assigned to a specific gene was then used as a representative proxy for gene fitness [14, 31]. (Figure 2b).

Mapping these z‐scores across a panel of genes previously identified as essential segregated them from the remaining genes in the genome for both conditions [12] (Figure 2c, Figure S3a). Specifically, the z‐scores of established core essential genes, such as those encoding ribosomal proteins and, core polymerase subunits exhibited the most pronounced depletion in both MM‐glc and MM‐muc (Figure S3b, c). This validates the negative fitness effects induced by CRISPRi‐mediated repression of essential genes. To quantitatively evaluate the temporal dynamics of the genetic screen, Pearson correlation coefficients were calculated across P_1_, P_3_, and P_F_ passages. This analysis revealed a progressive divergence over sequential passages in both intra‐passage and condition‐specific manners, reflecting the cumulative impact of CRISPRi‐mediated interference on strain fitness (Figure 2d). These shifts were highly reproducible between biological replicates (Figure S4), confirming that the observed shifts derive from selection rather than stochastic noise.

### 2.4 Comparative Gene Fitness Landscapes in Mucin and Glucose

Comparative analysis of gene‐level fitness z‐scores between MM‐glc and MM‐muc identified 1,256 genes with significant condition‐dependent divergence (*p*‐val < 0.05, Mann–Whitney U test). This suggests that these genes contribute to fitness in at least one condition through the passage (Table S4). Based on k‐means clustering, these genes were categorized into ten independent clusters (Figure 3a, Table S4). Functional enrichment analysis in each cluster revealed core essential processes, including ribosome [GO:0003735; bth03010], RNA binding [GO:0003723], and translation [GO:0006412], were enriched in clusters 1 and 2, consistent with their fundamental role in cellular viability.

**FIGURE 3:**
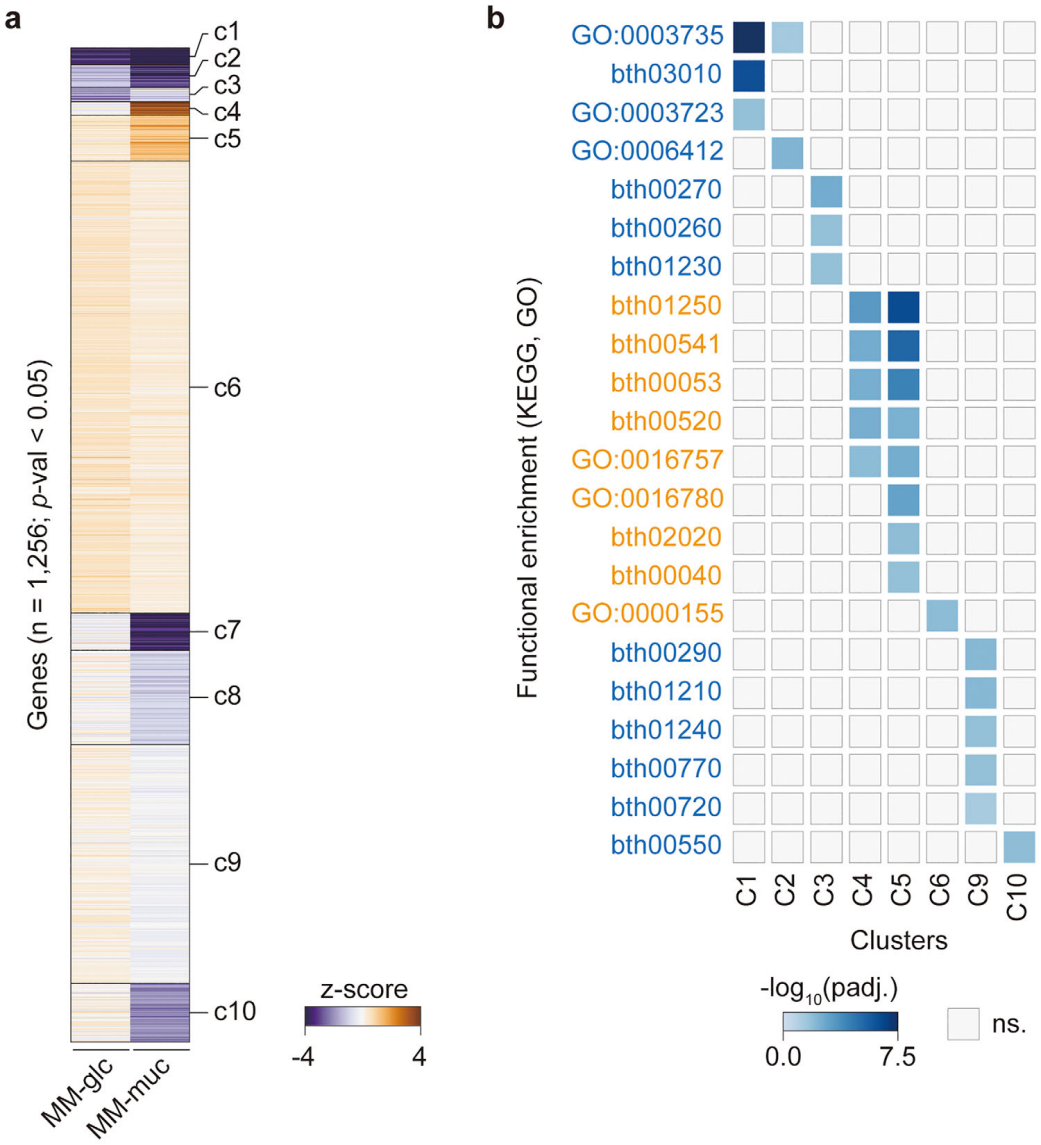
Comparison of CRISPRi fitness landscapes and functional enrichment networks of *B. thetaiotaomicron* in MM‐glc versus MM‐muc. (a) Hierarchical clustering of gene‐level fitness z‐scores comparing growth in MM‐glc to MM‐muc. The heatmap displays 1,256 genes exhibiting significant condition‐dependent fitness divergence (*p*‐val < 0.05, Mann–Whitney U test), partitioned into 10 distinct clusters (c1–c10). (b) Functional enrichment analysis of the corresponding gene clusters using Kyoto Encyclopedia of Genes and Genomes (KEGG) pathways and Gene Ontology (GO) terms. Categories with blue and orange hue denote mean z‐score lower or higher than 0 in the MM‐muc condition, respectively.

Beyond these core functions, several metabolic pathways exhibited condition‐specific fitness profiles. For example, cluster 3, in which genes tended to show lower z‐scores in MM‐glc (Table S4), was enriched in amino acid biosynthesis genes [bth01230] particularly those involved in cysteine and, methionine [bth00270], as well as glycine, serine, and threonine metabolism [bth00260] (Figure 3b). Gene‐level analysis further revealed condition‐specific fitness dependencies in amino acid metabolism in *B. thetaiotaomicron*, as consistent with previous findings (Text S1, Table S5) [12].

Notably, genes in cluster 4 and 5 exhibited mucin‐specific increases in z‐scores upon repression, indicating that their expression imposes a condition‐dependent fitness cost during growth on mucin. These clusters were enriched for functions related to sugar biosynthesis and glycosyl transferase activity [bth01250], biosynthesis of various nucleotide sugars [bth00541], amino sugar and nucleotide sugar metabolism [bth00520], glycosyltransferase activity [GO:0016757] (Figure 3b). Since most of these genes map to CPS loci (Table S4), we reasoned that capsule expression can hinder the initial access of bulky mucin glycans to the cell surface, implicating surface accessibility as a key determinant of mucin utilization fitness.

### 2.5 CPS Expression Imposes Fitness Cost in Mucin

Clusters 4 and 5 comprised nearly all genes (75 out of 76) that are dispensable in MM‐muc (z‐score > 1; *p*‐val < 0.05) (Figure 4a). The z‐score distribution across P_1_, P_3_, and P_F_ passages demonstrated a progressive fitness advantage for these repressed mutants in MM‐muc compared to MM‐glc (Figure 4b). Functional analysis within these clusters predominantly identified structural glycosyltransferases, export machinery, and regulatory genes belonging to several of the eight recognized *B. thetaiotaomicron* CPS loci (Figure 4c). To determine if this in vitro fitness advantage was reflected at the transcriptional level, we referred to RNA‐Seq expression profiling in MM‐glc and MM‐muc conducted in our previous study [26]. Notably, the CPS loci that incurred a fitness cost in MM‐muc, specifically CPS1, 3, 4, and 5, exhibited higher median expression than the other loci (Figure 4d), suggesting an active expression of the corresponding surface structures.

**FIGURE 4:**
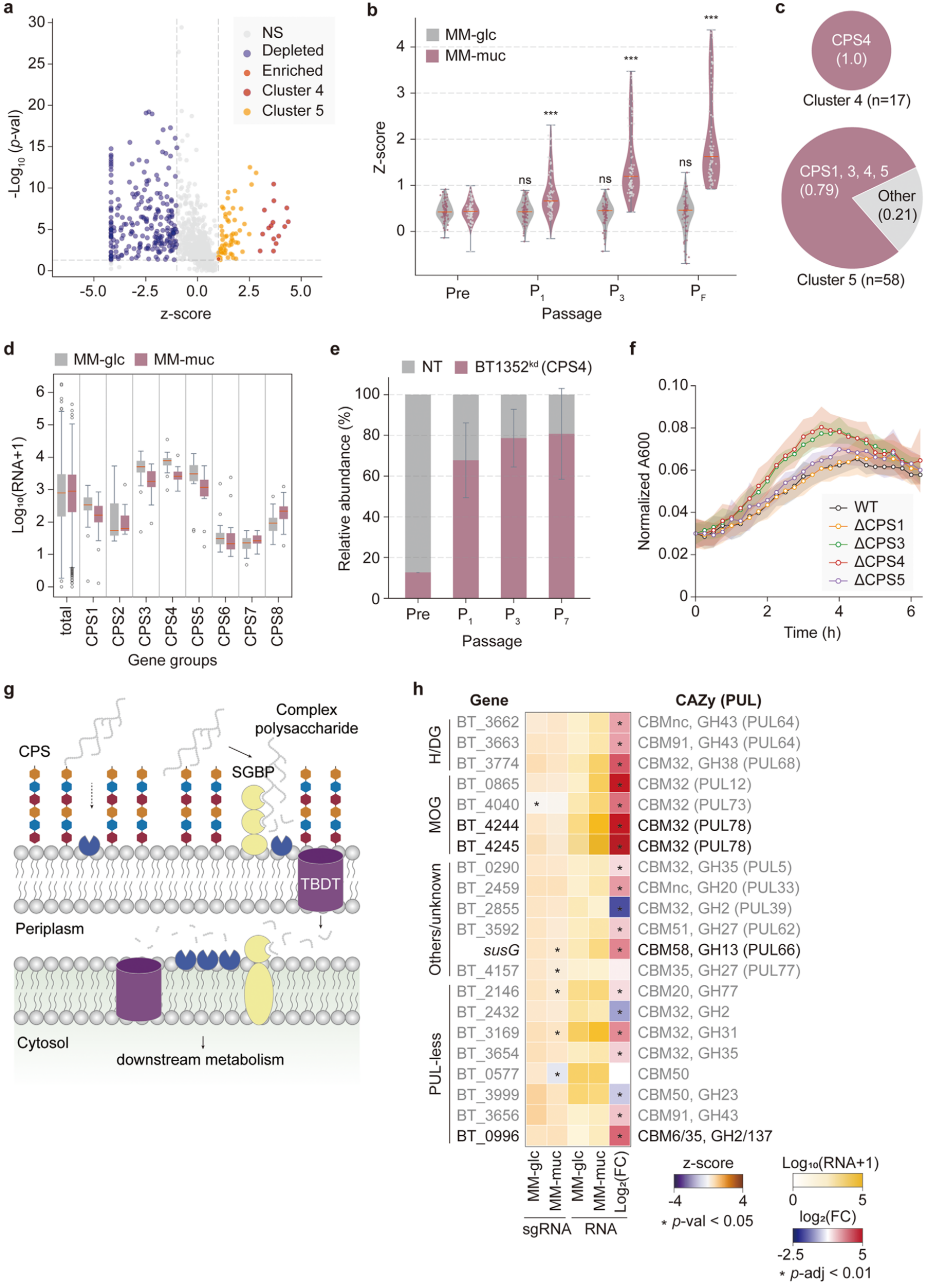
Enrichment of capsular polysaccharides reveals selected CPS‐associated fitness costs during growth on MM‐muc. (a) Volcano plot of genome‐wide CRISPRi fitness screens highlighting the profound enrichment of genes within clusters 4 and 5 during growth in minimal medium supplemented with MM‐muc. (b) *z*‐score distribution of the genes comprising clusters 4 and 5 across the pre‐culture (Pre) and sequential passages in MM‐glc and MM‐muc. (c) Compositional pie charts demonstrating gene composition of clusters 4 and 5 overwhelmingly dominated by structural genes mapping to the CPS biosynthetic loci. (d) Transcriptomic expression profiles of the eight annotated CPS loci in wild‐type *B. thetaiotaomicron* cultured in MM‐glc versus MM‐muc. (e) In vitro competition assay tracking the relative abundance of a targeted CPS4 knockdown mutant (BT_1352^kd^) against a non‐targeting (NT) control strain over successive passages in MM‐muc. (f) Growth profile of the wild‐type and specific deletion strains (ΔCPS1, ΔCPS3, ΔCPS4, ΔCPS5) cultured in MM‐muc. Data points represent the mean, and shaded regions indicate the standard deviation of biological triplicates. (g) Mechanistic schematic illustrating the CPS‐mediated steric hindrance hypothesis. CPS layer physically occludes access of structurally massive mucin to cellular membrane. Outer membrane‐anchored SGBP facilitates membrane localization of mucin via glycan recognition and binding. (h) Heatmap displaying encoding predicted glycan‐binding proteins containing CBM domains that are significantly changed in terms of either fitness (z‐score with *p*‐val < 0.05; Mann‐Whitney U) or expression (DEGs with *padj* < 0.01). CBM‐family assignments are based on CAZy annotations and primary biochemical literature (in gray), unless explicitly indicated as experimentally characterized (in black). Asterisks (*) indicates statistical significance. Abbreviations: H/DG—host or dietary glycans, MOG—mucin *O*‐glycans.

To experimentally validate these fitness inferences, we performed an in vitro co‐culture competition using a CPS4 knockdown strain (BT_1352^kd^), which exhibited the highest z‐score in MM‐muc (Table S4). We observed a progressive increase in its relative abundance, rising from ∼10–15% in the pre‐culture to ∼65–70% after the first mucin passage, and reaching ∼80% by later passages (Figure 4e). This demonstrates a substantial advantage under mucin selection. Furthermore, targeted deletions of entire CPS loci altered growth dynamics in MM‐muc, where both ΔCPS3 and ΔCPS4 mutants reached higher cell density in MM‐muc compared to the wild type, whereas differences for ΔCPS1 and ΔCPS5 were negligible (Figure 4f, Figure S5a, b), corroborating previous findings that CPS3 and CPS4 are the dominant surface structures expressed in vitro [32, 33]. These growth phenotypes are concordant with the inferred fitness scores, confirming that the metabolic impact of gene repression is also mirrored in complete deletion mutants. Together, these results demonstrate that capsule loci are not functionally equivalent, and that expressing specific capsules imposes a pronounced metabolic burden during growth on mucin.

Intriguingly, although CPS1, 3, 4, and 5 were downregulated in MM‐muc relative to MM‐glc (Figure 4d, Figure S6), their fitness cost remained largely neutral in MM‐glc (Table S4). This was validated experimentally, as the growth of both knockdown and knockout strains in MM‐glc remained largely neutral or marginally deleterious (Figure S5b–f). This suggests that factors beyond simple protein synthesis drive the observed fitness defects in MM‐muc. Mechanistically, the data support a “surface‐accessibility trade‐off” model. Previous studies have shown that the bacterial polysaccharide capsule can act as a physical diffusion barrier, restricting the access of complex carbohydrates to outer membrane nutrient acquisition machinery [17]. In *B. thetaiotaomicron*, this machinery comprises SusD‐like proteins, additional surface glycan‐binding proteins, surface‐associated CAZymes, and TonB‐dependent transporters. Some of these proteins additionally contain CBM domains that facilitate substrate recognition and binding [15, 16, 17, 18], where inactivation of the CBM domain compromises substrate binding activity [17]. In the current context, capsule synthesis likely reduces the effective reach of bulky mucin glycans at the outer membrane, thereby impairing their engagement by PUL‐associated sensing and uptake (Figure 4g).

As expected, RNA‐Seq analysis revealed significant induction of several genes encoding CBM‐containing proteins during growth in MM‐muc (Figure 4h), together with numerous SusC/SusD‐like and other TonB‐dependent transport systems (Figure S7). In particular, we observed strong induction of genes encoding predicted glycan‐binding proteins with CBM32 domains, which have been implicated in the recognition and binding of host *O*‐glycan motifs [34]. Among them, BT_4244, a well‐characterized mucinase that preferentially cleaves mucin *O*‐glycans, and BT_4245, a cognate SGBP for mucosal glycan recognition [18, 21, 35], were among the most highly up‐regulated genes (Figure 4h) [5]. Similarly, we observed a marked induction in BT_0996, which contains CBM6 and 35 from a rhamnogalacturonan II‐degrading enzyme [36], and *susG* that binds to starch at the outer membrane [17]. Other predicted CBMs containing genes, such as CBM51 that bind host blood group antigens and related epitopes [37], and CBM91 with activity on xylo‐oligosaccharides [38], respectively, were also up‐regulated. However, it should be noted that several CBM‐like β‐sandwich domains have been classified as bacterial immunoglobulin‐like modules and shown to function in catalytic‐domain stabilization, or active‐site assembly, instead of autonomous glycan binding [39, 40]. Thus, the result for proposed glycan‐binding proteins with predicted CBMs may reflect increased deployment of multidomain glycan‐processing proteins, instead of their autonomous glycan capture.

Despite this widespread upregulation, however, no individual glycan‐binding and sensing machineries exhibited a significant fitness contribution in MM‐muc (Figure 4h, Figure S7). This pattern seems to reflect functional overlap among multiple surface‐binding, catalytic, and transport components. Nevertheless, this coordinated overexpression is consistent with a surface‐accessibility model where specific CPS states hinder the access of bulky mucin to outer‐membrane capture systems. Taken together, these results suggest that while individual glycan‐binding machineries may be redundant, their collective upregulation constitutes an essential adaptive strategy for efficient mucin utilization.

### 2.6 Functional Robustness of CAZymes

Just as *B. thetaiotaomicron* deploys a repertoire of glycan‐recognition and binding proteins for initial substrate capture, our gene‐level fitness analysis revealed extensive functional robustness within the downstream catalytic machinery (Figure 5a, Figure S8). Specifically, various GH families, which catalyze the hydrolysis of glycosidic linkages in polysaccharides [41], exhibited neutral fitness z‐scores (|z‐score| < 1.0) in MM‐muc. One exception is BT_0771, which encodes a GH13 glucan‐branching enzyme, showing a MM‐muc specific fitness defect (Figure S9a, b). Previous Tn‐Seq results also showed depletion of BT_0771 across different carbon sources [12], suggesting an essential nature of BT_0771 in strain fitness (Figure S9c, Text S2). When mapping these GH families to their predicted enzymatic cleavage sites on the complex mucin *O*‐glycan structure (Figure 5b) [41], we observed that genes within key mucin‐targeting families were generally upregulated in MM‐muc compared to MM‐glc (Figure 5c). However, despite this strong condition‐specific induction, the targeted repression of these individual GH genes resulted in negligible fitness defects.

**FIGURE 5:**
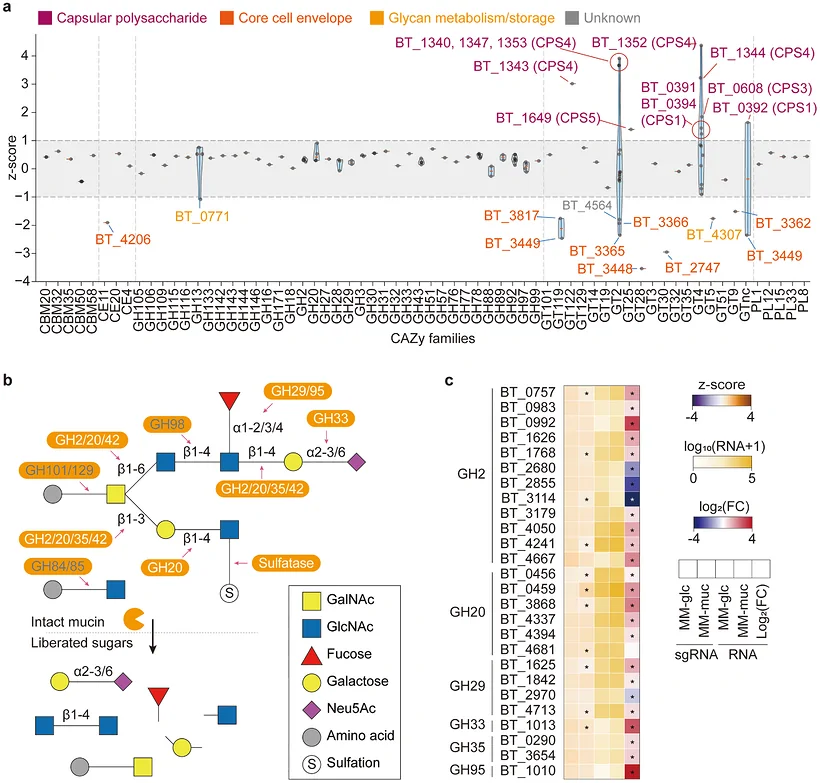
Fitness landscapes of CAZy family enzymes and pathways for mucin glycan degradation. (a) Distribution of z‐scores for genes categorized by CAZy families statistically significant in MM‐muc. Violin plots show the spread of z‐scores for individual genes within each family. Genes with |z‐score| > 1.0 are highlighted. (b) Schematic model of intact mucin *O*‐glycan degradation by *B. thetaiotaomicron*. GHs encoded in *B. thetaiotaomicron* are marked in white, those absent are marked in gray. (c) Heatmaps comparing the fitness contributions and transcriptional expression of specific mucin‐targeting GH genes that are significantly changed in terms of either fitness (z‐score with *p*‐val < 0.05; Mann–Whitney U) or expression (DEGs with *padj* < 0.01). Asterisk (*) statistical significance.

We surmise that these near‐neutral fitness scores in GH knockdown strains can be ascribed to two non‐exclusive factors, including (i) extensive functional complementarity within the *Bacteroides* glycan‐degrading machinery and (ii) the structural complexity of mucin itself. First, *B. thetaiotaomicron* allocates a significant portion of its genome to diverse PULs and their embedded CAZymes [9]. This elaborate gene repertoire provides CAZyme isozymes with overlapping substrate specificities that may complement against the loss of any single hydrolase [6, 41]. Second, while degrading complex glycans such as mucin, *B. thetaiotaomicron* co‐expresses arrays of distinct CAZymes to harvest a diverse pool of monomers for downstream metabolism [4, 5, 42]. Consequently, the simultaneous hydrolysis of multiple glycan branches may generate a ‘sufficient’ nutrient supply to support growth, even when specific partner enzymes are absent—though strict metabolic bottlenecks do exist in some cases [43]. Taken together, these findings suggest substantial apparent robustness to individual CRISPRi perturbations within the extracellular mucin‐processing network under the conditions tested.

Taken together, gene‐level fitness profiling across saccharolytic CAZymes and cognate PULs revealed that repression of most individual genes produced neutral or statistically insignificant fitness effects during growth on MM‐muc. This apparent lack of fitness changes did not reflect transcriptional inactivity. Rather, transcriptomic analysis based on *B. thetaiotaomicron* iModulon framework [26] showed a broad induction of CAZyme‐associated gene modules, including multiple PUL iModulons, in MM‐muc relative to MM‐glc (Text S3, Figures S10 and S11, Table S6), consistent with previous reports of extensive mucin‐responsive PUL activation in *B. thetaiotaomicron* [5, 44]. Such disconnect between broad transcriptional induction and minimal single‐gene fitness defects suggests that mucin foraging is supported by a highly distributed and functionally redundant extracellular degradation network, such that repression of individual CAZyme components is insufficient to compromise overall fitness.

### 2.7 Fitness and Expression Profiles of Downstream Metabolic Pathways of Mucin‐Constituent Sugars

We next investigated whether the intracellular pathways assimilating liberated monosaccharides exhibit a matching fitness architecture. Once complex host glycans are cleaved into constituent monosaccharides such as GlcNAc, GalNAc, fucose, and galactose [23], these sugars are channeled through dedicated catabolic pathways into central carbon metabolism (Figure 6a–d) [7].

**FIGURE 6:**
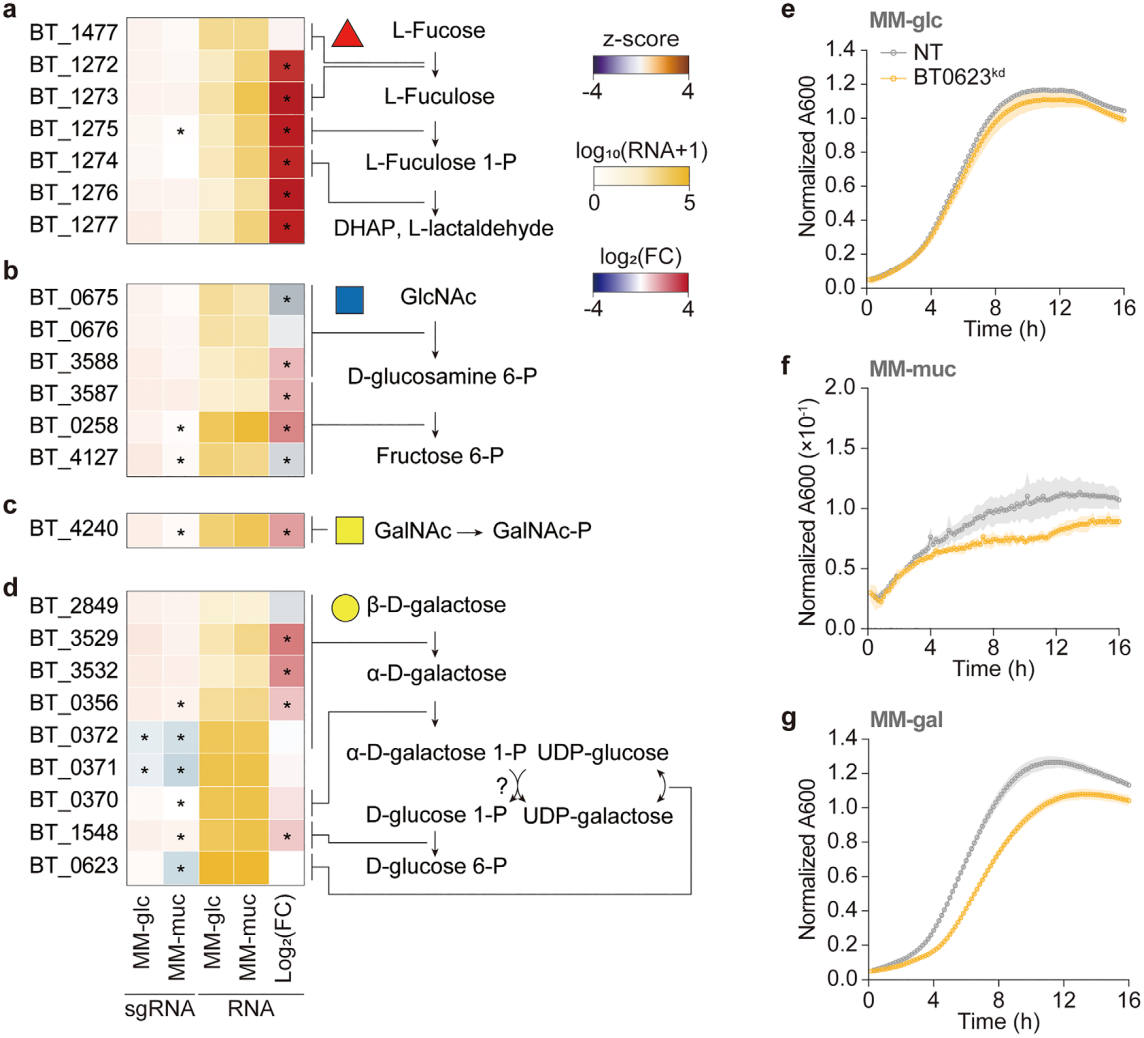
Fitness and expression profile of metabolic pathways of mucin constituent sugars. (a) Heatmaps detailing the fitness and transcriptional expression of genes involved in the degradation and utilization of specific liberated sugars (GlcNAc, L‐Fucose, GalNAc, and galactose) during growth in MM‐glc and MM‐muc. Asterisks (*) denote statistically significant fitness effects (*p*‐val < 0.05), while “ns” indicates not significant. (b‐d) Growth curve comparison between NT control and a knockdown strain for BT_0623 (BT0623^kd^) in (b) MM‐glc, (c) MM‐muc, and (d) MM‐galactose. Data points represent the mean, and shaded regions indicate the standard deviation of biological triplicates.

Consistent with this framework, the pathways downstream of mucin‐constituent sugars displayed distinct combinations of transcriptional activation and genetic requirement. First, transcriptomic analysis showed a generally concordant upregulation of catabolic pathways in MM‐muc. In particular, the fucose‐responsive BT_1272‐1277 operon, which governs fucose isomerization and phosphorylation [2, 24], exhibited significant upregulation compared to MM‐glc (Figure 6a). However, most genes in this module showed minimal to no fitness defects, suggesting that the transcriptional induction of the fucose module does not necessarily translate into a growth bottleneck in a pooled screen.

In the case of GlcNAc utilization, the metabolic pathway exhibited divergent transcriptional responses among specific isozymes (Figure 6b). For example, among genes encoding *N*‐acetylglucosamine‐6‐phosphate deacetylase, BT_0675 and BT_0676 were downregulated, whereas BT_3588 was upregulated. Similarly, for the downstream glucosamine‐6‐phosphate deaminase isozymes, BT_3587 and BT_0258 were induced, while BT_4127 was repressed (Figure 6b). This pattern closely mirrors previously reported expression profiles during GlcNAc‐specific induction [33], highlighting the primary involvement of specific isozymes in GlcNAc metabolism. Regarding gene fitness, all genes remained neutral upon knockdown, indicating functional redundancy among these metabolic genes.

In contrast, the galactose utilization branch exhibited clear condition‐specific functional dependencies. Multiple genes within the predicted Leloir pathway, including BT_0371 (glucose/galactose transporter), BT_0372 (aldose 1‐epimerase), and BT_0623 (UDP‐glucose 4‐epimerase), displayed significant negative fitness effects in MM‐muc (Figure 6d). This finding is consistent with a previous report identifying these three genes as core fitness determinants during carbohydrate utilization [11]. Although their transcript levels were higher in MM‐muc than in MM‐glc, these differences were not statistically significant in our dataset. Previous studies have reported mucin‐specific induction of these genes, which showed that their expression is responsive to host glycan availability [33, 44]. Together, these results suggest that the galactose utilization machinery may be maintained at a sufficient basal level to support growth across conditions but becomes specifically required when mucin‐derived sugars serve as the primary carbon input. Consistent with this, earlier work has linked BT_0370–BT_0371 and BT_0623 to galactose utilization during growth on mucin [44], highlighting that galactose, which is the most abundant constituent sugar in mucin [23], serves as a functionally important carbon source for *B. thetaiotaomicron*.

To experimentally validate these fitness predictions, we focused on BT_0623, which selectively yielded negative z‐scores in MM‐muc within the galactose catabolic pathway (Figure 6d). Relative to the NT control, the BT_0623 knockdown strain (BT0623^kd^) showed a reproducible growth defect in MM‐muc but not in MM‐glc (Figure 6e, f, Figure S12a, b). This phenotype became more pronounced when galactose was provided as the sole carbon source (Figure 6d, Figure S12c), establishing a direct role for BT_0623 in mucin‐ and galactose‐dependent growth. Together, these data show a functional conservation and specific metabolic bottlenecks required for growth in the presence of mucin.

### 2.8 Comparative Analysis of In Vitro and In Vivo CRISPRi Screens

Next, to determine whether the mucin‐dependent fitness landscape observed in vitro is retained in the host, we performed pooled in vivo CRISPRi screens during murine colonization (Figure 7a, Text S4, Figure S13). We identified genes showing significant z‐score changes (*p*‐val < 0.05; Mann–Whitney U) in the colon relative to the input library (Tables S3 and S7). Among genes with significant fitness effects in either the colon or MM‐muc, fitness scores were strongly correlated between the two environments (Pearson R = 0.78, 95% CI = 0.76–0.80), exceeding the correlation between the colon and MM‐glc (Figure 7b, Figure S14). Consistent with this global trend, pathway‐level analysis showed that MM‐muc more closely recapitulated the colon fitness profile across most KEGG biological processes than did MM‐glc (Figure S15a). However, specialized functional categories associated with capsule biosynthesis and glycan acquisition, including CPS, PUL and CAZyme groups, showed greater divergence between MM‐muc and the colon than between MM‐glc and the colon (Figure S15b, c). Furthermore, quadrant‐level analysis further showed that shared depletion in MM‐muc and the colon was dominated by core translational functions, whereas discordant quadrants were enriched for glycosyltransferase‐ and locus‐specific PUL/CAZyme/CPS patterns, indicating that specialized surface and glycan‐utilization functions diverge between mucin‐defined growth and the host colon environment (Figure 7c; Table S8, Text S5). Together, these results indicate that MM‐muc captures broad host‐relevant physiological constraints but only partially reflects specialized surface‐associated and glycan‐processing functions shaped by the intestinal niche.

**FIGURE 7:**
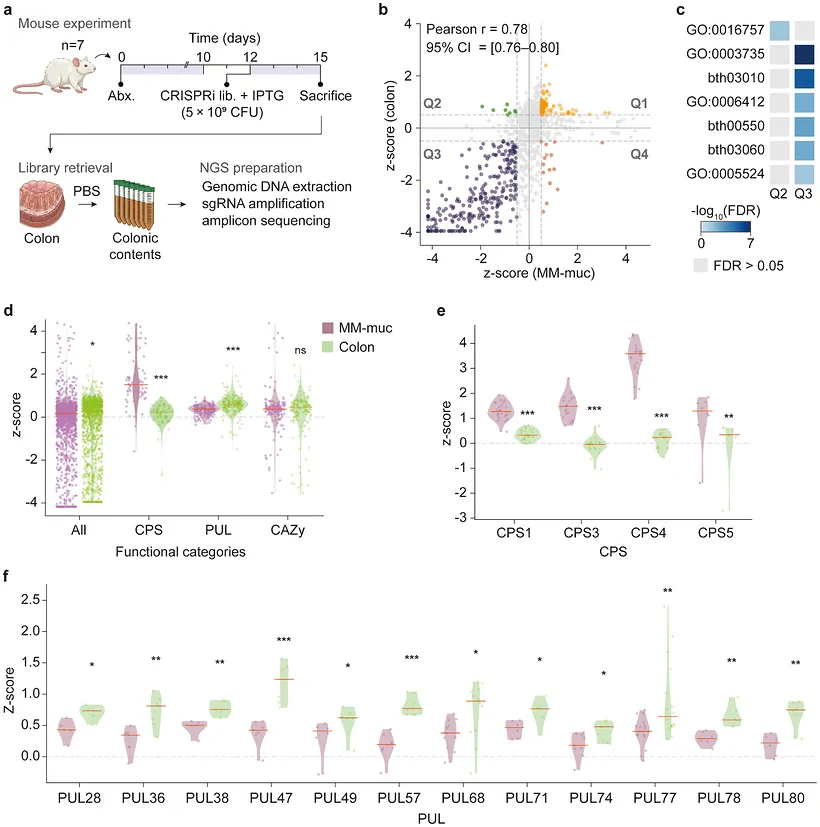
In vivo fitness profiling of *B. thetaiotaomicron* and correlation with in vitro mucin‐dependent growth. (a) Schematic overview and timeline of the in vivo mouse colonization model. Arrows indicate the time point at which specific interventions were administered. (b) Scatter plot comparing CRISPRi fitness z‐scores obtained from in vitro growth in MM‐muc against in vivo colonization in the murine colon. (c) Heatmap detailing functional enrichment analysis for genes categorized in Q2 and Q3 from panel b. (d) Violin plots comparing the distribution of fitness z‐scores across distinct functional gene categories between the MM‐muc and colon environments. (e‐f) Violin plots in sub‐categories of (e) CPS and (f) PUL. Red horizontal lines denote the median values. Statistical differences between the two conditions are indicated (* *p*‐val < 0.05, *** *p*‐val < 0.001, ns = not significant).

### 2.9 Divergent Fitness Profile of Mucin Utilization Networks In Vitro and In Vivo

Next, we analyzed gene fitness profiles across the specialized categories CPS, PUL and CAZy. Notably, category‐specific divergence was especially evident for genes belonging to CPSs and PULs, and to a lesser extent for CAZy‐associated genes (Figure S15b, c). Consistently, CPS and PUL fitness distributions differed significantly between MM‐muc and the colon, whereas CAZy genes did not (Figure 7d). Detailed subcategory analysis further revealed that the pronounced fitness advantage of repressing CPS genes in MM‐muc, which we suggest as a phenomenon driven by the cost of capsule expression in vitro (Figure 4), was not evident in the colon environment (Figure 7e, Table S9). This disparity highlights a trade‐off in capsular expression, where we speculate that the apparent metabolic cost of CPS synthesis is counterbalanced by host‐imposed selective pressures, such as immune evasion [32], phage sensitivity [45], biofilm formation [46], and niche competition [47], all of which are irrelevant in a strictly controlled in vitro MM‐muc. This corroborates previous observations where CPS4 inactivation impairs in vivo fitness [10, 11, 48], likely through these host‐relevant traits [32, 45, 47]. Additionally, the difference between our CRISPRi results (CPS4 neutral in vivo) and prior knockout phenotypes (CPS4 lethal in vivo) may reflect the distinct consequences of partial knockdown versus complete gene loss [49].

CRISPRi strains targeting genes in several PULs, including PUL47, PUL68, and PUL77, were relatively enriched in the colon compared to MM‐muc, indicating that repression of these loci is more favorable in vivo (Figure 7f). PUL47 and PUL68 are induced by host glycans, whereas PUL77 is associated with utilization of the plant polysaccharide rhamnogalacturonan‐I (RG‐I) [5, 50]. These patterns are consistent with *B. thetaiotaomicron* encountering a nutritionally heterogeneous and competitive gut environment, where expressing certain PULs may be energetically unfavorable [10, 11]. Consistent with this result, repression of the PUL77‐encoded GH2 exo‐β‐1,4‐galactosidases BT_4151 and BT_4156, which remove residual galactan substituents from RG‐I, resulted in relative enrichment in the colon (Table S9). This contrasts with a prior in vivo fitness screen, where BT_4151 showed gradual depletion during gut colonization, whereas BT_4156 remained neutral throughout [12]. These divergent fitness outcomes further highlight that fitness contribution of PUL‐associated genes is contingent upon the nutrition context of the surrounding environment [11].

By contrast, several other GHs were depleted in vivo, including BT_4187, a GH28 protein genetically implicated in RG‐I utilization; BT_4305, a predicted GH57 α‐glucan‐active enzyme; and BT_0771, a predicted GH13 1,4‐α‐glucan branching enzyme, as consistent with their reported fitness profiles [11, 12]. Notably, although prior mutagenesis studies showed that both BT_4187 and BT_4149, which encode GH28 proteins, are important for type I RG‐I utilization [12, 51], these two genes showed strikingly different in vivo fitness profiles, where BT_4187 was depleted (z‐score: ‐1.97), while BT_4149 was enriched (z‐score: 1.67) (Table S9). This contrast suggests that BT_4187 may support a varied in vivo function beyond that of RG‐I utilization or that its role is less easily complemented than that of BT_4149.

Nonetheless, most PUL‐associated genes remained dispensable at the individual level in vivo. Of the 866 PUL‐associated genes [5], only twelve retained negative z‐scores in the colon, and only two (BT_3461 and BT_4723) showed marked depletion (z‐score < ‐1; *p*‐val < 0.05) (Table S9). This suggests that the loss of individual glycan‐acquisition functions can often be compensated for presumably because the afflicted mutants still maintain sufficient enzymatic capacity to access a redundant repertoire of glycans [48]. Together, these patterns suggest that fitness changes in PULs and cognate CAZymes may arise from shifts in substrate prioritization or access to host‐modified glycans, features not fully captured by MM‐muc alone [10, 11].

Lastly, the fitness profiles of downstream sugar catabolic pathways were largely conserved, with the galactose utilization branch remaining the most negatively impacted (particularly for BT_0371 and BT_0372), while BT_0623 showed a milder defect in vivo (Table S9). This conservation suggests that while PULs and CAZymes offer flexibility, downstream galactose utilization remains a constant determinant of fitness across both mucin‐rich and host‐associated conditions, as reported previously.

## 3 Discussion

In this study, we integrated genome‐wide CRISPRi and RNA‐Seq to leverage system‐scale datasets for reconstruction of the mucin utilization network in *B. thetaiotaomicron*. The flow of our analysis spans from glycan access at the surface layer, degradation by CAZymes, to downstream sugar assimilation network (Figure 1). Then, we further compared in vitro mucin‐dependent fitness profiles with in vivo datasets to distinguish core determinants of mucin utilization from host‐environment‐specific adaptations.

At the surface layer, the enrichment of CPS‐repressed strains, together with targeted competition and deletion phenotypes, showed that capsule‐associated traits, although beneficial in vivo [32, 45, 46, 47], can impose context‐dependent fitness costs during mucin utilization under controlled in vitro conditions. Current data are consistent with a model in which specific CPS limit access to mucin‐derived nutrients at the cell surface, consistent with evidence that surface architecture influences complex glycan capture [17, 34]. However, as this study does not directly demonstrate steric occlusion, these observations should be framed as functional inferences rather than definitive mechanisms.

At the extracellular interface, many mucin‐induced glycan‐binding and hydrolytic proteins, including selected CBM‐containing CAZymes and SusC, D‐like proteins made little detectable contribution to fitness upon perturbation despite clear transcriptional activation. This transcript–fitness decoupling is consistent with the architecture of *Bacteroides* glycan‐utilization systems, in which the associated proteins can act on overlapping substrates, thereby functionally complementing for individual gene perturbations [52]. However, it should be noted that this apparent redundancy may be specific to mucin utilization and are not generalized to other polysaccharides or host‐derived glycans. For example, complex arabinogalactan utilization can depend on an initiating surface endo‐β‐1,3‐galactanase, chondroitin‐sulfate catabolism contains a rate‐determining glucuronyl hydrolase, and heparin/heparan‐sulfate degradation requires surface and periplasmic enzymes with distinct substrate specificities [53, 54, 55]. These studies illustrate that PUL‐mediated glycan utilization can contain non‐redundant, pathway‐constraining enzymatic steps. By contrast, the downstream metabolic layer appeared less uniformly complemented. Among the pathways for mucin‐constituent sugars, the galactose branch showed pronounced condition‐specific fitness defects, where the repression of BT_0623 impaired growth on both mucin and galactose. A plausible interpretation is that while the liberation of mucin‐derived sugars is distributed across a partially redundant network, intracellular assimilation involves narrower metabolic bottlenecks. However, this conclusion remains measured, while our data identify galactose catabolism as a key determinant in this setting, they do not establish it as the sole dominant carbon route during mucin‐supported growth.

The comparison with murine colonization is most informative where it diverges from the MM‐muc landscape. The strong enrichment of CPS‐repressed strains in MM‐muc remained largely neutral in vivo. This suggests that capsule fitness is shaped less by carbon utilization efficiency alone than by ecological contingencies absent in vitro. The outer colonic mucus layer is a spatially distinct niche rather than a simple reservoir of mucin glycans. Previous work has shown that bacterial behavior in this compartment is influenced by localization, resource partitioning, and microbial competition [11, 13]. In that context, the in vitro CPS phenotype likely reflects the nutrient‐access cost of capsule expression, which manifests only when its host‐associated benefits become irrelevant. In contrast, the relative enrichment of several PULs in vivo compared with MM‐muc suggests that colonization rewards substrate breadth and rapid metabolic switching over exclusive specialization for mucin. Our results support the view of the host gut as a heterogeneous and fluctuating nutrient landscape, in which host glycans, dietary polysaccharides, and community‐derived breakdown products coexist.

Several limitations define the scope of this study. First, CRISPRi measures partial repression rather than a complete loss of function. Consequently, the resulting phenotypes may underestimate the contributions of genes with fitness effects emerge only below a critical activity threshold, which may obscure the distinction between true functional redundancy from incomplete knockdown. This likely explains the neutral fitness scores of CPS4‐repressed strains, where CPS4 deletions typically show defects [10, 11, 48] and a relatively ‘mild’ fitness defect in BT_0623^kd^, despite its status as core fitness determinant in carbohydrate metabolism [11]. Second, pooled CRISPRi screen can leave ‘cheaters’ undefeated despite loss of a critical function. For example, BT1636, an S1_20 3‐*O*‐sulfogalactose sulfatase essential for utilization of highly sulfated colonic mucin *O*‐glycans and in vivo fitness [43], showed no significant fitness effect in the current screen and in previous pooled screening studies [11, 12]. The overwhelming majority of neighboring strains retain BT1636 activity and may collectively remove terminal sulfate groups, potentially allowing BT1636‐deficient cells to access processed glycans or downstream metabolites. Although such trans‐complementation was not directly tested, it provides a plausible mechanism by which a non‐redundant extracellular activity could be underestimated in pooled functional‐genetic screens.

An additional caveat concerns with the biochemical composition of mucin used in this study—MM‐muc contained commercially processed porcine gastric mucin, which differs from native colonic mucin in composition and *O*‐glycan repertoire [56]. These differences may influence the relative accessibility and abundance of specific glycan structures and therefore limit the extent to which individual MM‐muc fitness determinants can be directly extrapolated to native colonic mucin. Nevertheless, the substantial concordance between the MM‐muc and murine colon fitness landscapes indicates that this experimentally tractable mucin condition captures a meaningful subset of host‐relevant physiological constraints. Lastly, MM‐muc lacks the spatial complexity, mixed nutrient inputs, and microbial antagonism of the host niche and other environmental variables that shape colonization. Future studies should focus on defining which extracellular mucin‐acquisition functions remain limiting under more host‐relevant conditions, such as organoid models [57], and on clarifying how capsule expression modulates nutrient access and fitness across distinct gut environments.

## 4 Materials and Methods

### 4.1 Bacterial Strains and Culture Conditions

The *B. thetaiotaomicron* VPI‐5482 strain (ATCC 29148) was sourced from the Korean Collection for Type Cultures. Routine cultivation of *B. thetaiotaomicron* strains was carried out in brain heart infusion‐supplemented broth (BHIS) or Columbia Blood Agar (MBcell, no. MB‐C2146) supplemented with 5% defibrinated sheep blood (MBcell, no. MB‐S1876). One liter of BHIS liquid medium contains the following components: 37 g of BHI powder (BD Bacto, no. 237500), 5 g of yeast extract (BD Bacto, no. 212750), 0.5 g/L L‐cysteine hydrochloride monohydrate (TCI, no. C0517), 0.2 mM L‐histidine (Sigma, no. H6034), and 1.9 µM hemin (Sigma, no. 51280), with the pH adjusted to 8. The menadione solution (1 µg/mL, Sigma, no. M5625) was dissolved in absolute ethanol (Merck, no. 100983). L‐cysteine, L‐histidine‐hemin solution, and menadione were filter‐sterilized using a Minisart 0.2 µm syringe filter (Sartorius, no. 16534K) and added to the media immediately prior to cell culture. Minimal medium contains 100 mM of KH_2_PO_4_, 15 mM of NaCl, 8.5 mM of (NH_4_)_2_SO_4_, 0.5 g/L L‐cysteine hydrochloride monohydrate, 0.2 mM L‐histidine, 1.9 µM hemin, 50 µM CaCl_2_, 100 µM MgCl_2_, 1.4 µL FeSO_4_∙7H2O, 1 µg/mL menadione, and 5 ng/mL vitamin B12. All carbon sources including glucose (Sigma‐Aldrich, no. G7021), galactose (Sigma, no. G5388), and mucin from porcine stomach (Sigma‐Aldrich, no. M1778) were added to minimal medium with a final concentration 0.5% (w/v), unless stated otherwise. Appropriate antibiotics and inducer, including erythromycin (25 µg/mL; Sigma‐Aldrich, no. E5389), gentamicin (200 µg/mL; Sigma‐Aldrich, no. G1264), and Isopropyl β‐D‐1‐thiogalactopyranoside (100 µM; Sigma‐Aldrich, no. I5502) were used as required. Cell cultures were incubated in 150 mL serum bottles, with up to 50 mL of culture medium, purged with N_2_/CO_2_ (90:10) gas at 80 kPa. Competition assay was performed by mixing two heterogeneous populations at a set ratio in 50 mL of minimal medium containing porcine mucin as the sole carbon source, at an initial OD_600_ ∼ 0.02. Cultures were incubated at 37°C, 200 rpm for 6 h, then serially passaged to a fresh MM‐muc at 100‐fold dilution, for a total of 6 passages. The initial passage, and the passage 2, 4 and 6 were prepared for NGS sequencing. Growth profiling was carried out in an Epoch 2 plate reader (BioTek) at 37°C with shaking (807 rpm, double orbital) over a 24‐h period, and OD_600_ absorbance was recorded every 10 min. For plate reader experiments, porcine mucin was added at 0.2% (w/v) to minimize optical interference due to turbidity. All plate‐reader datasets are normalized with a corresponding blank, pathlength‐corrected, and baseline‐shifted to the set initial OD_600_. Specific growth rate (µ) was determined between five consecutive timepoints that yield the highest rate along the log phase of growth, as calculated by the standard equation. The *E. coli* S17‐1 λ pir strain (ATCC BAA‐2428), used as a conjugation helper strain, was kindly provided by Professor Pil Kim at the Catholic University of Korea. The *E. coli* strains were cultured aerobically at 37°C in Luria–Bertani broth or on agar plates supplemented with 100 µg/mL ampicillin (Sigma‐Aldrich, no. A0166).

### 4.2 Vector Construction

All plasmids used in this study are listed in Table S10. Plasmid cloning and propagation were carried out in PIR1 One Shot *E. coli* (Invitrogen, no. C101010) as per the manufacturer's guidelines. Phusion High‐Fidelity DNA Polymerase was employed for high‐fidelity amplifications and DNA sequencing. All oligonucleotides were synthesized by Macrogen. DNA purification was carried out using QIAquick PCR Purification Kit (Qiagen, no. 28104), QIAquick Gel Extraction Kit (Qiagen, no. 28704), and DNA‐spin Plasmid DNA Purification Kit (iNtRON Biotechnology, no. 17098).

The sgRNA expression vector was developed using the pMM710 plasmid (Addgene, no. 68543), which is based on the pNBU2 backbone and incorporates the intN2 tyrosine integrase along with attN sites. These features facilitate site‐specific recombination with one of two attBT sites, BT_t70 and BT_t71, enabling precise integration into the *B. thetaiotaomicron* genome. The sgRNA cassettes, comprising a promoter, N20 spacer, and scaffold, were assembled using primer pairs that incorporate BpiI type II restriction enzyme sites at their 5' and 3' termini, respectively (Full sequence in Table S11). After PCR amplification and purification, the sgRNA cassettes and the pMM553 backbone vector were each subjected to digestion with BpiI enzyme (Thermo Scientific, no. FD1014), generating compatible ends for subsequent ligation. The digested sgRNA cassettes were then inserted into the linearized pMM553 vector backbone using T4 DNA ligase (Invitrogen, no. 15224017), following the manufacturer's instructions.

CPS knockout strains were designed as described previously [20]. Construction of gene knockout vectors targeting four CPS loci were designed based on pLGB13 counter selection vector [58]. Specifically, homology arms were cloned into pLGB13 and propagated in *E. coli* S17‐1 λpir. The CRISPRi construct was transferred to *B. thetaiotaomicron* via conjugation and selected using appropriate selection markers.

### 4.3 Design Consideration for sgRNA Oligomer Library

The sgRNAs were designed to target the 5' end of every 100 bp segment of the open reading frame (ORF). In cases where no sgRNA target site was available within a 100‐bp window, the nearest available target site from the preceding segment was chosen. To ensure effective transcription inhibition, sgRNAs were designed to bind to the coding strand of the target ORFs, following the methodology reported in a previous study [28]. Off‐target sequences that did not meet the specified criteria in a previous study were excluded [59]. The final library consisted of 55,378 sgRNAs designed to target at roughly 100‐bp intervals of the ORF sequences.

### 4.4 Synthesis of Oligomer Library and Assembly of Plasmid Library

The sgRNA oligomers were synthesized and cloned into a NBU‐2‐based plasmid vector. The plasmid library was received as a glycerol cell stock from GENEWIZ. The pooled plasmid library was cultured, and plasmids were subsequently purified using a commercial plasmid extraction kit, following the manufacturer's instructions.

### 4.5 Transformation of sgRNA Plasmid Library to Helper Strain for Conjugation

The conjugation of the bacterial host was initiated by electroporating the purified sgRNA plasmid library into the helper strain *E. coli* S17‐1 λpir. To prepare electrocompetent *E. coli* S17‐1 λpir cells, an overnight culture was grown in LB medium. This culture was then transferred into 50 mL of fresh LB medium with an initial OD_600_ of 0.05. The cells were allowed to grow until the mid‐log phase, which was reached when the OD_600_ was between 0.4 and 0.6. The cells were pelleted by centrifugation at 4,000 × *g* for 10 min at 4°C and washed twice with ice‐cold 10% (v/v) glycerol. After washing, the cells were resuspended in 100 µL of ice‐cold 10% glycerol. The prepared competent cells were immediately frozen in liquid nitrogen and stored at ‐80°C until transformation.

For electroporation, 500 ng of the purified sgRNA plasmid library was added to 100 µL of electrocompetent *E. coli* S17‐1 λpir cells. The mixture was gently mixed and transferred into 1 mm electroporation cuvettes. Electroporation was performed using a Bio‐Rad MicroPulser Electroporator, following the recommended protocol for *E. coli* transformation, with a voltage setting of 1.8 kV and using 0.1 cm cuvettes. Following electroporation, the cells were immediately resuspended in 900 µL of SOC medium and incubated for 1 h at 37°C to allow for recovery. After recovery, the cells were serially diluted and spread onto LB agar plates containing ampicillin to select for transformed cells. The transformation efficiency was evaluated by counting the number of colonies and determining the necessary number of plates to achieve the desired number of colonies. Once colonies were visible, they were scraped and resuspended in LB medium supplemented with ampicillin. A 50% (v/v) glycerol solution was added in an equal volume to create a 25% (v/v) glycerol stock, which was stored at ‐80°C. All procedures following the scraping of cells were performed on ice to minimize any potential loss of viability. A total of 4.5 × 10^6^ colonies were obtained, yielding 81‐fold coverage of the designed sgRNA library.

### 4.6 Conjugation of Bacterial Hosts

The sgRNA library was integrated into the *B. thetaiotaomicron* genome through conjugation, facilitated by the helper strain harboring the sgRNA plasmid library. This process was carried out as described by previous protocols [60]. Briefly, *B. thetaiotaomicron* strains containing an integrated dCas9 system were cultured overnight and diluted into BHIS medium to an OD_600_ of 0.05. The cultures were allowed to grow to early exponential phase (OD_600_ between 0.2 and 0.4) before harvesting. The harvested *B. thetaiotaomicron* cells were mixed with the *E. coli* S17‐1 λpir cells, which contained the sgRNA plasmid library, at a 1:5 (v/v) ratio. Two‐hundred microliters of the mixture was pooled onto CBA plates and incubated aerobically at 37°C for 20 h in a standing incubator to allow conjugative mating to occur. After the incubation, the cell lawn was scraped from the plates and resuspended in 20 mL of BHIS liquid medium. Ten milliliters of cell suspensions were plated on BHIS agar containing erythromycin (25 µg/mL) and gentamicin (200 µg/mL) in square plates (SPL Life Sciences, no. 10245). These plates were incubated in an anaerobic chamber (20% CO_2_, 4% H_2_, and balanced nitrogen) at 37°C for 48 h. Following incubation, colonies were harvested and resuspended in BHIS liquid medium containing erythromycin. The resuspended cells were aliquoted into cryovials with the addition of 50% (v/v) glycerol to create 20% (v/v) glycerol stocks, which were stored at ‐80°C. Prior to storage, the cryovials were maintained in the anaerobic chamber to allow for proper gas exchange. The individual stocks from the colonies were combined into a unified cell stock, with the final stock composition proportional to the number of colonies originally formed.

In this experiment, approximately 10^5^ colonies were obtained per conjugation round. A total of 35 conjugation rounds were performed, resulting in approximately 3 × 10^6^ colonies. Seven conjugations were carried out each day over the span of five days, resulting in approximately 3.5 × 10^6^colonies. This procedure generated an sgRNA library with an average coverage of 63‐fold.

### 4.7 In Vitro CRISPRi Screening

The main cultures of *B. thetaiotaomicron* CRISPRi library were maintained in MM‐glc, or in MM‐muc. For MM‐glc, main culture underwent seven serial passages, starting from an initial OD_600_ of 0.05 and growing to a final OD_600_ of 1.0, encompassing 4–5 generations per passage for a total of approximately 30 generations. For MM‐muc, the main culture underwent six serial passages, each initiated at an OD_600_ of 0.02 and incubated for 6 h before dilution and transfer. All cultures were supplemented with erythromycin (25 µg/mL) and induced with IPTG (100 µM) to promote dCas9 expression. Main cultures were also performed in triplicate and maintained in 150 mL serum bottles containing 50 mL of culture media, purged with a N_2_/CO_2_ (90:10) gas mixture at 80 kPa, and incubated at 37 °C with shaking at 200 rpm.

### 4.8 In Vivo CRISPRi Screening

In vivo CRISPRi screening experiments were conducted using a CRISPRi library of *B. thetaiotaomicron* in male C57BL/6J mice. Mice received a 10‐day antibiotic treatment regimen to facilitate colonization of the orally gavaged bacteria. Specifically, mice were provided drinking water supplemented with 1 g/L metronidazole (Sigma‐Aldrich, no. M1547) and 0.625 mg/mL ciprofloxacin (Sigma‐Aldrich, no. 17850), sweetened with 3% sucrose (Sigma‐Aldrich, no. 84100), for the first seven days. This was followed by oral gavage of metronidazole at 100 mg/kg once every 24 h and continued ciprofloxacin administration in the drinking water for the subsequent three days. Following a two‐day antibiotic‐free period, mice received a single oral gavage of 5 × 10^9^ CFU of engineered *B. thetaiotaomicron* cultured in BHIS medium supplemented with 100 µM IPTG until the log phase. From the day of inoculation (day 0) until euthanasia (day 3), mice were fed with drinking water supplemented with 25 mM IPTG. Colon tissues were harvested by flushing luminal contents with phosphate‐buffered saline (PBS).

### 4.9 DNA Extraction

Cells were collected by centrifugation at 4,000 × *g* for 10 min at 4°C. Genomic DNA was extracted using the Wizard Genomic DNA Purification Kit (Promega, no. A1125) according to the manufacturer's instructions. DNA quality was assessed by measuring the A260/A280 ratio, ensuring it was above 1.8, and by performing agarose gel electrophoresis. DNA concentration was determined using a NanoDrop 2000 spectrophotometer (Thermo Scientific).

### 4.10 NGS Library Preparation and Sequencing

Genomic DNA (gDNA) was extracted from the conjugant cell stock using the Wizard Genomic DNA Purification Kit, with concentration measured using the Qubit 1× dsDNA BR assay kit (Invitrogen, no. Q33265). Gel electrophoresis was used to detect any gDNA shearing. A nested PCR strategy was used to specifically amplify the sgRNA sequences inserted into the *B. thetaiotaomicron* genome, avoiding amplification from the S17‐1 helper strain. In the first step of PCR amplification, primers specific to both the *B. thetaiotaomicron* genome and the sgRNA were used to amplify the sgRNAs integrated into the genome. The PCR reaction mixture consisted of 10 µL of 5× Q5 Reaction Buffer, 1 µL of 10 mM dNTPs, 1 µL each of BT_F and sgRNA_R primers (10 µM stock), 0.5 µL of Q5 High‐Fidelity DNA polymerase, 1 µL of 10× SYBR Green solution, 25 ng of gDNA, and nuclease‐free water to make up a total volume of 50 µL. The PCR conditions included an initial denaturation at 98°C for 3 min, followed by cycles of 98°C for 10 s, 65°C for 30 s, and 72°C for 2 min, with a final extension at 72°C for 2 min, and storage at 4°C. The size of the PCR product was verified by gel electrophoresis, with the expected product size being 3,353 bp. PCR products were purified using a commercial kit, with gel extraction performed if non‐specific bands were detected. The second round of nested PCR amplified the portions of the sgRNA sequence required for sequencing. The reaction mixture contained 1 µL each of UDi5_F1 and UDi7_R1 primers, 25 µL of HotStarTaq Master Mix, 1 µL of 10× SYBR Green solution, 50 ng of purified PCR product, and nuclease‐free water to a total volume of 50 µL. The PCR conditions included an initial denaturation at 95°C for 15 min, followed by 30 cycles of 94°C for 30 s, 54°C for 30 s, and 72°C for 30 s, with a final extension at 72°C for 10 min, and storage at 4°C. Gel electrophoresis of the second PCR amplicons revealed the expected product size of 301 bp. The amplicons were purified using a commercial kit, and the concentration and size distribution of the sequencing library were analyzed using a Qubit 1× dsDNA HS assay kit (Invitrogen, no. Q33230) and a D1000 ScreenTape (Agilent, no. 5067–5582) with D1000 reagents (Agilent, no. 5067–5583) on the Agilent 2200 TapeStation system. The final sequencing library was quantified by qPCR using the KAPA Library Quantification Kit (Roche, no. KK4824) as an additional quality control measure. Sequencing was carried out on the MiSeq System with a 150‐cycle single‐end run, incorporating 10% PhiX control as recommended by the manufacturer. The sequencing data were processed using the CLC Genomics Workbench, where low‐quality reads and adapter sequences were trimmed. The promoter sequence (CAATTGGGCTACCTTTTTTTTGTTTTGTTTGCAATGGTTAATCTATTGTTAAAATTTAAAGTTTCACTTGAACTTTCAAATAATGTTCTTATATTTGCAG) and scaffold sequences (GTTTTAGAGCTAGAAATAGCAAGTTAAAATAAGGCTAGTCCGTTATCAACTTGAAAAAGTGGCACCGAGTCGGTGC) were removed, leaving only the spacer sequences (N20 sequences). The final data were analyzed by counting the reads corresponding to each unique spacer sequence, which were then mapped to the designed sgRNAs for downstream analysis.

### 4.11 Data Processing for CRISPRi Datasets

Read mapping: Low‐quality reads and adapter sequences were trimmed using the CLC Genomics Workbench with a limit = 0.05 and a maximum of two ambiguous nucleotides. The promoter sequence (CAATTGGGCTACCTTTTTTTTGTTTTGTTTGCAATGGTTAATCTATTGTTAAAATTTAAAGTTTCACTTGAACTTTCAAATAATGTTCTTATATTTGCAG) and scaffold sequence (GTTTTAGAGCTAGAAATAGCAAGTTAAAATAAGGCTAGTCCGTTATCAACTTGAAAAAGTGGCACCGAGTCGGTGC) were removed, resulting in the retention of the spacer sequence. Each distinct spacer sequence was matched to its corresponding sgRNA to determine the read count for each individual sgRNA.

Fitness score calculation: The fitness of each sgRNA was assessed by comparing its abundance in the selected library to that in the initial library using z‐score values. Specifically, gene fitness (***Fitness***_***gene***_) was calculated as the 75th percentile of the sgRNA fitness values (***Fitness***_***sgRNA***_) for all sgRNAs targeting a specific gene [31]. To control for differences in sequencing depth between samples, the read count of each sgRNA was normalized by the total read counts within each sample, as shown in Equation (1):

(1) $$\mathrm{Frequenc}y_{\mathrm{sgRNA}}=\frac{\mathrm{read}\;\mathrm{count}}{\sum_{i=1}^{n}\mathrm{read}\;\mathrm{count}}$$

Subsequently, a pseudocount of 10^−6^ was added to the normalized read counts to avoid the issues associated with log transformation of zero‐counts while minimally impacting the data distribution. The normalized read counts were then log‐transformed using base 10, as shown in Equation (2):

(2) $$F^{\prime }=\log_{10}\left({\text{Frequency}}_{\text{sgRNA}}+\varepsilon \right),\varepsilon =10^{-6}$$

The z‐score for each sgRNA (***Fitness***_***sgRNA***_) was computed by subtracting the mean log‐transformed frequency of all sgRNAs in the initial library from the log‐transformed frequency of the selected library. This result was then divided by the standard deviation of the log‐transformed frequencies of all sgRNAs in the initial library, as represented in Equation (3):

(3)

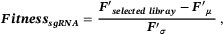


***F′***_***μ***_ = average of the log‐transformed frequencies for all sgRNA in initial library,

***F′***_***σ***_ = standard deviation of the log‐transformed frequencies for all sgRNA in initial library

***Fitness***_***gene***_ was determined as the 75th percentile of the fitness values for all sgRNAs targeting the corresponding gene, as outlined in Equation (4):

(4) $$\;\mathrm{Fitnes}s_{\mathrm{gene}}=75^{\mathrm{th}}\;\mathrm{percentile}\;\mathrm{of}\;\left\{Z_{\mathrm{sgRNA}1},\;Z_{\mathrm{sgRNA}2},\;Z_{\mathrm{sgRNA}3},\text{…}\;Z_{\mathrm{sgRNAn}}\right\}$$

Using the 75th percentile to represent gene fitness offers a statistically robust measure compared to calculating fitness based on the log_2_fold‐change of the selected library relative to the initial library [14]. Additionally, the percentile approach effectively excludes ineffective spacers and mitigates the impact of sgRNA off‐target effects. This method ensures that the fitness values do not overestimate or underestimate the actual sgRNA read counts and accurately quantify both depleted and enriched fitness effects, as reported in previous studies [31].

To identify genes exhibiting significant changes in ***Fitness***_***gene***_, a Mann–Whitney U test was performed. This test compared the ***Fitness***_***sgRNA***_ values of sgRNAs targeting each gene in the selected library with those in the initial library. A total of 1,256 genes were significantly changed in the final passages of MM‐glc or MM‐muc. Genes were grouped into 10 clusters based on Silhouette score analysis using unsupervised k‐means clustering. GO and KEGG pathway enrichment analysis was performed using ClusterProfiler [61], and a Benjamini–Hochberg–corrected *p*‐val < 0.05 was considered significant.

Root mean square error (RMSE) was used as a descriptive measure of absolute discrepancy between paired gene‐level fitness z‐scores, with lower RMSE indicating closer correspondence to the in vivo fitness profile. For each functional subcategory, RMSE was defined as with lower values indicating closer correspondence to the in vivo profile, as shown in Equation 5.

(5) $$\sqrt{\frac{1}{n}\sum_{i=1}^{n}{\left(z_{i},\;\mathrm{condition}-z_{i},\;\mathrm{colon}\right)}^{2}}$$

RMSE was calculated separately for KEGG, CPS, PUL and CAZy subcategories using genes with non‐missing z‐scores that are statistically significant in either one of the compared conditions; only subcategories containing at least five genes were retained. For CAZy analysis, comma‐delimited annotations were split so that the first and second annotated families were counted independently.

### 4.12 Data Processing for RNA‐Seq Datasets

Low‐quality reads were trimmed using the following parameters—quality limit = 0.05; maximum ambiguous nucleotides = 2, and mapped to the reference genome with the following parameters—mismatch cost = 2; insertion cost = 3; deletion cost = 3; length fraction = 0.9; similarity cost = 0.9. Uniquely mapped reads were retrieved and normalized using the Bioconductor package DESeq2 with default parameters [62]. Transcript per million was calculated using an in‐house Python code with Sequence Alignment/Map files. To infer iModulon activities, the log_10_‐transformed expression levels of downloaded RNA‐Seq data, with in‐batch normalization to MM‐glc, were projected onto the BtModulome [26], using the pymodulon package [63].

### 4.13 Animals

All procedures were carried out in accordance with the guidelines approved by the Institutional Animal Care and Use Committee at the Korea Research Institute of Bioscience and Biotechnology (Approval Number: KRIBB‐AEC‐22253). Six‐week‐old male C57BL/6J mice were procured from The Jackson Laboratory (Bar Harbor) and housed at the Korea Research Institute of Bioscience and Biotechnology. The animals were kept in a controlled environment with a 12‐h light/dark cycle and had free access to Teklad Global 18% Protein Rodent Diet (Envigo, no. 2018S) and water. Prior to the start of the experiments, the mice were allowed a two‐week acclimatization period to adapt to the housing conditions.

## Author Contributions

B.‐K.C. conceived and supervised the study. K.K., J.L., M.K., D.C., and B.‐K.C. designed the experiments. K.K., J.L., and M.K. performed the experiments. K.K., J.L., M.K., and D.C. analyzed the data, K.K., J.L., M.K., S.S.Y., D.C., and B.‐K.C. wrote and edited the manuscript. All authors have read and approved the final manuscript.

## Conflicts of Interest

The authors declare no conflicts of interest.

## Supporting information




**Supporting File 1**: advs78118‐sup‐0001‐TableS1.xlsx.

**Supporting File 2**: advs78118‐sup‐00010‐TableS9.xlsx.

**Supporting File 3**: advs78118‐sup‐00011‐TableS10.xlsx.

**Supporting File 4**: advs78118‐sup‐0002‐SuppMat.pdf.

**Supporting File 5**: advs78118‐sup‐0003‐TableS2.xlsx.

**Supporting File 6**: advs78118‐sup‐0004‐TableS4.xlsx.

**Supporting File 7**: advs78118‐sup‐0005‐TableS5.xlsx.

**Supporting File 8**: advs78118‐sup‐0006‐TableS6.xlsx.

**Supporting File 9**: advs78118‐sup‐0007‐TableS6.xlsx.

**Supporting File 10**: advs78118‐sup‐0008‐TableS7.xlsx.

**Supporting File 11**: advs78118‐sup‐0009‐TableS8.xlsx.

**Supporting File 12**: advs78118‐sup‐0012‐TableS11.xlsx.

## Data Availability

The data that support the findings of this study are openly available in EMBL Nucleotide Sequence Database at https://www.ebi.ac.uk/ena/browser/, reference number ERA36082103.

## References

[1] A. G. Wexler and A. L. Goodman , “An Insider's Perspective: Bacteroides as a Window Into the Microbiome,” Nature Microbiology 2, no. 5 (2017): 17026, 10.1038/nmicrobiol.2017.26. PMC5679392 28440278

[2] P. C. Kashyap , A. Marcobal , L. K. Ursell , et al., “Genetically Dictated Change in Host Mucus Carbohydrate Landscape Exerts a Diet‐Dependent Effect on the Gut Microbiota,” Proceedings of the National Academy of Sciences 110, no. 42 (2013): 17059–17064, 10.1073/pnas.1306070110. PMC3800993 24062455

[3] J. L. Sonnenburg , J. Xu , D. D. Leip , et al., “Glycan Foraging *in Vivo* by an Intestine‐Adapted Bacterial Symbiont,” Science 307, no. 5717 (2005): 1955–1959, 10.1126/science.1109051. 15790854

[4] M. Boutard , T. Cerisy , P.‐Y. Nogue , et al., “Functional Diversity of Carbohydrate‐Active Enzymes Enabling a Bacterium to Ferment Plant Biomass,” PLoS Genetics 10, no. 11 (2014): 1004773, 10.1371/journal.pgen.1004773. PMC4230839 25393313

[5] E. C. Martens , H. C. Chiang , and J. I. Gordon , “Mucosal Glycan Foraging Enhances Fitness and Transmission of a Saccharolytic Human Gut Bacterial Symbiont,” Cell Host & Microbe 4, no. 5 (2008): 447–457, 10.1016/j.chom.2008.09.007. PMC2605320 18996345

[6] A. Rogowski , J. A. Briggs , J. C. Mortimer , et al., “Glycan Complexity Dictates Microbial Resource Allocation in the Large Intestine,” Nature Communications 6, no. 1 (2015): 7481, 10.1038/ncomms8481. PMC4491172 26112186

[7] D. A. Ravcheev and I. Thiele , “Comparative Genomic Analysis of the Human Gut Microbiome Reveals a Broad Distribution of Metabolic Pathways for the Degradation of Host‐Synthetized Mucin Glycans and Utilization of Mucin‐Derived Monosaccharides,” Frontiers in Genetics 8 (2017): 111, 10.3389/fgene.2017.00111. PMC5583593 28912798

[8] J. Shin , J.‐R. Noh , D.‐H. Chang , et al., “Elucidation of *Akkermansia muciniphila* Probiotic Traits Driven by Mucin Depletion,” Frontiers in Microbiology 10 (2019): 1137, 10.3389/fmicb.2019.01137. PMC6538878 31178843

[9] P. Lapébie , V. Lombard , E. Drula , N. Terrapon , and B. Henrissat , “ *Bacteroidetes* Use Thousands of Enzyme Combinations to Break Down Glycans,” Nature Communications 10, no. 1 (2019): 2043, 10.1038/s41467-019-10068-5. PMC6499787 31053724

[10] M. S. Kennedy , M. Zhang , O. DeLeon , et al., “Dynamic Genetic Adaptation of *Bacteroides thetaiotaomicron* During Murine Gut Colonization,” Cell Reports 42, no. 8 (2023): 113009, 10.1016/j.celrep.2023.113009. PMC10528517 37598339

[11] M. Wu , N. P. McNulty , D. A. Rodionov , et al., “Genetic Determinants of in vivo Fitness and Diet Responsiveness in Multiple Human Gut *Bacteroides* ,” Science 350, no. 6256 (2015): aac5992, 10.1126/science.aac5992. PMC4608238 26430127

[12] H. Liu , A. L. Shiver , M. N. Price , et al., “Functional Genetics of Human Gut Commensal *Bacteroides thetaiotaomicron* Reveals Metabolic Requirements for Growth Across Environments,” Cell Reports 34, no. 9 (2021): 108789, 10.1016/j.celrep.2021.108789. PMC8121099 33657378

[13] M. S. Desai , A. M. Seekatz , N. M. Koropatkin , et al., “A Dietary Fiber‐Deprived Gut Microbiota Degrades the Colonic Mucus Barrier and Enhances Pathogen Susceptibility,” Cell 167, no. 5 (2016): 1339–1353.e21, 10.1016/j.cell.2016.10.043. PMC5131798 27863247

[14] W. Jiang , P. Oikonomou , and S. Tavazoie , “Comprehensive Genome‐Wide Perturbations Via CRISPR Adaptation Reveal Complex Genetics of Antibiotic Sensitivity,” Cell 180, no. 5 (2020): 1002–1017.e31, 10.1016/j.cell.2020.02.007. PMC7169367 32109417

[15] G. Déjean , K. Tamura , A. Cabrera , et al., “Synergy Between Cell Surface Glycosidases and Glycan‐Binding Proteins Dictates the Utilization of Specific Beta(1,3)‐Glucans by Human Gut Bacteroides,” MBio 11, no. 2 (2020): e00095–20, 10.1128/mBio.00095-20. PMC7157763 32265336

[16] J. M. Grondin , K. Tamura , G. Déjean , D. W. Abbott , and H. Brumer , “Polysaccharide Utilization Loci: Fueling Microbial Communities,” Journal of Bacteriology 199, no. 15 (2017): e00860–16, 10.1128/JB.00860-16. PMC5512228 28138099

[17] E. A. Cameron , K. J. Kwiatkowski , B.‐H. Lee , B. R. Hamaker , N. M. Koropatkin , and E. C. Martens , “Multifunctional Nutrient‐Binding Proteins Adapt Human Symbiotic Bacteria for Glycan Competition in the Gut by Separately Promoting Enhanced Sensing and Catalysis,” MBio 5, no. 5 (2014): e01441–14, 10.1128/mBio.01441-14. PMC4173775 25205092

[18] D. A. Ndeh , S. Nakjang , K. J. Kwiatkowski , et al., “A *Bacteroides thetaiotaomicron* Genetic Locus Encodes Activities Consistent With Mucin *O*‐Glycoprotein Processing and *N*‐Acetylgalactosamine Metabolism,” Nature Communications 16, no. 1 (2025): 3485, 10.1038/s41467-025-58660-2. PMC11992087 40216766

[19] E. C. Martens , R. Roth , J. E. Heuser , and J. I. Gordon , “Coordinate Regulation of Glycan Degradation and Polysaccharide Capsule Biosynthesis by a Prominent Human Gut Symbiont,” Journal of Biological Chemistry 284, no. 27 (2009): 18445–18457, 10.1074/jbc.M109.008094. PMC2709373 19403529

[20] T. E. Rogers , N. A. Pudlo , N. M. Koropatkin , et al., “Dynamic Responses of *Bacteroides thetaiotaomicron* During Growth on Glycan Mixtures,” Molecular Microbiology 88, no. 5 (2013): 876–890, 10.1111/mmi.12228. PMC3700664 23646867

[21] S. Nakjang , D. A. Ndeh , A. Wipat , D. N. Bolam , and R. P. Hirt , “A Novel Extracellular Metallopeptidase Domain Shared by Animal Host‐Associated Mutualistic and Pathogenic Microbes,” PLoS ONE 7, no. 1 (2012): 30287, 10.1371/journal.pone.0030287. PMC3267712 22299034

[22] A. Benjdia , E. C. Martens , J. I. Gordon , and O. Berteau , “Sulfatases and a Radical S‐Adenosyl‐L‐Methionine (Adomet) Enzyme Are Key for Mucosal Foraging and Fitness of the Prominent Human Gut Symbiont, *Bacteroides thetaiotaomicron* ,” Journal of Biological Chemistry 286, no. 29 (2011): 25973–25982, 10.1074/jbc.M111.228841. PMC3138274 21507958

[23] A. P. Gunning , A. R. Kirby , C. Fuell , C. Pin , L. E. Tailford , and N. Juge , “Mining the “Glycocode”—Exploring the Spatial Distribution of Glycans in Gastrointestinal Mucin Using Force Spectroscopy,” The FASEB Journal 27, no. 6 (2013): 2342–2354, 10.1096/fj.12-221416. PMC3659345 23493619

[24] L. V. Hooper , J. Xu , P. G. Falk , T. Midtvedt , and J. I. Gordon , “A Molecular Sensor That Allows a Gut Commensal to Control Its Nutrient Foundation in a Competitive Ecosystem,” Proceedings of the National Academy of Sciences 96, no. 17 (1999): 9833–9838, 10.1073/pnas.96.17.9833. PMC22296 10449780

[25] J. Xu , M. K. Bjursell , J. Himrod , et al., “A Genomic View of the Human‐*Bacteroides thetaiotaomicron* Symbiosis,” Science 299, no. 5615 (2003): 2074–2076, 10.1126/science.1080029. 12663928

[26] K. Kim , D. Choe , S. C. Kim , et al., “Transcriptional Regulatory Networks of the Human Gut Symbiont *Bacteroides thetaiotaomicron* Are Uncovered Using Machine Learning,” Nucleic Acids Research 53, no. 20 (2025): gkaf1166, 10.1093/nar/gkaf1166. PMC12596196 41206038

[27] L. Cui , A. Vigouroux , F. Rousset , H. Varet , V. Khanna , and D. Bikard , “A CRISPRi Screen in *E. coli* Reveals Sequence‐Specific Toxicity of dCas9,” Nature Communications 9, no. 1 (2018): 1912, 10.1038/s41467-018-04209-5. PMC5954155 29765036

[28] L. S. Qi , M. H. Larson , L. A. Gilbert , et al., “Repurposing CRISPR as an RNA‐Guided Platform for Sequence‐Specific Control of Gene Expression,” Cell 152, no. 5 (2013): 1173–1183, 10.1016/j.cell.2013.02.022. PMC3664290 23452860

[29] D. Choe , E. Lee , Y. Song , et al., “CRISPRi Screening Reveals *E. coli′*s Anaerobic‐Like Respiratory Adaptations to Gentamicin: Membrane Depolarization by CpxR,” mSystems 10 (2025): 0035325. 10.1128/msystems.00353-25 PMC12282077 40521885

[30] T. Wang , C. Guan , J. Guo , et al., “Pooled CRISPR Interference Screening Enables Genome‐Scale Functional Genomics Study in Bacteria With Superior Performance,” Nature Communications 9, no. 1 (2018): 2475, 10.1038/s41467-018-04899-x. PMC6018678 29946130

[31] J. Shin , J. Bae , H. Lee , et al., “Genome‐Wide CRISPRi Screen Identifies Enhanced Autolithotrophic Phenotypes in Acetogenic Bacterium *Eubacterium limosum* ,” Proceedings of the National Academy of Sciences 120, no. 6 (2023): 2216244120, 10.1073/pnas.2216244120. PMC9963998 36716373

[32] N. T. Porter , P. Canales , D. A. Peterson , and E. C. Martens , “A Subset of Polysaccharide Capsules in the Human Symbiont *Bacteroides thetaiotaomicron* Promote Increased Competitive Fitness in the Mouse Gut,” Cell Host & Microbe 22, no. 4 (2017): 494–506.e8, 10.1016/j.chom.2017.08.020. PMC5830307 28966055

[33] D. Ryan , E. Bornet , G. Prezza , et al., “An Expanded Transcriptome Atlas for *Bacteroides thetaiotaomicron* Reveals a Small RNA that Modulates Tetracycline Sensitivity,” Nature Microbiology 9, no. 4 (2024): 1130–1144, 10.1038/s41564-024-01642-9. PMC10994844 38528147

[34] Y. Narimatsu , C. Pleguezuelos‐Manzano , D. Hornikx , et al., “Discovery of a Secreted *Bacteroides fragilis* Mucinase That Cleaves Mucins With Bis‐T O‐Glycans Through a Carbohydrate Binding Module‐Dependent Mechanism,” Gut Microbes 18, no. 1 (2026): 2644983, 10.1080/19490976.2026.2644983. PMC13003899 41856961

[35] V. Taleb , Q. Liao , Y. Narimatsu , et al., “Structural and Mechanistic Insights Into the Cleavage of Clustered *O*‐Glycan Patches‐Containing Glycoproteins by Mucinases of the Human Gut,” Nature Communications 13, no. 1 (2022): 4324, 10.1038/s41467-022-32021-9. PMC9325726 35882872

[36] F. Trovao , V. G. Correia , F. M. Lourenco , et al., “The Structure of a *Bacteroides thetaiotaomicron* Carbohydrate‐Binding Module Provides New Insight Into the Recognition of Complex Pectic Polysaccharides by the Human Microbiome,” Journal of Structural Biology: X 7 (2023): 100084. 10.1016/j.yjsbx.2022.100084 PMC9843283 36660365

[37] K. J. Gregg , R. Finn , D. W. Abbott , and A. B. Boraston , “Divergent Modes of Glycan Recognition by a New Family of Carbohydrate‐Binding Modules,” Journal of Biological Chemistry 283, no. 18 (2008): 12604–12613, 10.1074/jbc.M709865200. PMC2335362 18292090

[38] S. L. Pang , Y. Y. Wang , L. Wang , X. J. Zhang , and Y. H. Li , “The CBM91 Module Enhances the Activity of β‐Xylosidase/α‐L‐Arabinofuranosidase PphXyl43B From Paenibacillus Physcomitrellae XB by Adopting a Unique Loop Conformation at the top of the Active Pocket,” International Journal of Biological Macromolecules 266 (2024): 131275, 10.1016/j.ijbiomac.2024.131275. 38556222

[39] I. A. Kataeva , V. N. Uversky , J. M. Brewer , et al., “Interactions Between Immunoglobulin‐Like and Catalytic Modules in *Clostridium thermocellum* Cellulosomal Cellobiohydrolase CbhA,” Protein Engineering Design and Selection 17, no. 11 (2004): 759–769, 10.1093/protein/gzh094. 15596428

[40] H. Okano , E. Kanaya , M. Ozaki , C. Angkawidjaja , and S. Kanaya , “Structure, Activity, and Stability of Metagenome‐Derived Glycoside Hydrolase Family 9 Endoglucanase With an N‐Terminal Ig‐Like Domain,” Protein Science 24 (2015): 408–419. 10.1002/pro.2632 PMC4353366 25545469

[41] A. Bell and N. Juge , “Mucosal Glycan Degradation of the Host by the Gut Microbiota,” Glycobiology 31, no. 6 (2021): 691–696, 10.1093/glycob/cwaa097. PMC8252862 33043970

[42] D. Ndeh , A. Rogowski , A. Cartmell , et al., “Complex Pectin Metabolism by Gut Bacteria Reveals Novel Catalytic Functions,” Nature 544, no. 7648 (2017): 65–70, 10.1038/nature21725. PMC5388186 28329766

[43] A. S. Luis , C. Jin , G. V. Pereira , et al., “A Single Sulfatase Is Required to Access Colonic Mucin by a Gut Bacterium,” Nature 598, no. 7880 (2021): 332–337, 10.1038/s41586-021-03967-5. PMC9128668 34616040

[44] B. Liu , D. R. Garza , D. Gonze , et al., “Starvation Responses Impact Interaction Dynamics of Human Gut Bacteria *Bacteroides thetaiotaomicron* and *Roseburia intestinalis* ,” The ISME Journal 17, no. 11 (2023): 1940–1952, 10.1038/s41396-023-01501-1. PMC10579405 37670028

[45] N. T. Porter , A. J. Hryckowian , B. D. Merrill , et al., “Phase‐Variable Capsular Polysaccharides and Lipoproteins Modify Bacteriophage Susceptibility in *Bacteroides thetaiotaomicron* ,” Nature Microbiology 5, no. 9 (2020): 1170–1181, 10.1038/s41564-020-0746-5. PMC7482406 32601452

[46] N. Béchon , J. Mihajlovic , S. Vendrell‐Fernández , et al., “Capsular Polysaccharide Cross‐Regulation Modulates *Bacteroides thetaiotaomicron* Biofilm Formation,” MBio 11, no. 3 (2020): e00729–20, 10.1128/mBio.00729-20. PMC7315117 32576670

[47] D. Hoces , G. Greter , M. Arnoldini , et al., “Fitness Advantage of *Bacteroides thetaiotaomicron* Capsular Polysaccharide in the Mouse Gut Depends on the Resident Microbiota,” Elife 12 (2023): 81212, 10.7554/eLife.81212. PMC10014078 36757366

[48] A. L. Goodman , N. P. McNulty , Y. Zhao , et al., “Identifying Genetic Determinants Needed to Establish a Human Gut Symbiont in Its Habitat,” Cell Host & Microbe 6, no. 3 (2009): 279–289, 10.1016/j.chom.2009.08.003. PMC2895552 19748469

[49] H. Y. Lo , D. R. Long , E. A. Holmes , et al., “Transposon Sequencing Identifies Genes Impacting *Staphylococcus aureus* Invasion in a Human Macrophage Model,” Infection and Immunity 91 (2023): 0022823. 10.1128/iai.00228-23 PMC10580828 37676013

[50] E. C. Martens , E. C. Lowe , H. Chiang , et al., “Recognition and Degradation of Plant Cell Wall Polysaccharides by Two Human Gut Symbionts,” PLoS Biology 9, no. 12 (2011): 1001221, 10.1371/journal.pbio.1001221. PMC3243724 22205877

[51] A. S. Luis , J. Briggs , X. Zhang , et al., “Dietary Pectic Glycans Are Degraded by Coordinated Enzyme Pathways in Human Colonic *Bacteroides* ,” Nature Microbiology 3, no. 2 (2018): 210–219, 10.1038/s41564-017-0079-1. PMC5784806 29255254

[52] S. R. Schaus , C. Jin , G. Raba , et al., “Degradation of Mucin *O*‐Glycans by a Human Gut Symbiont Requires a Complex Enzyme Repertoire and Promotes Colonization,” preprint *bi* *oRxiv* March 26, 2026, 10.64898/2026.03.26.714468.

[53] A. Cartmell , J. Muñoz‐Muñoz , J. A. Briggs , et al., “A Surface Endogalactanase in *Bacteroides thetaiotaomicron* Confers Keystone Status for Arabinogalactan Degradation,” Nature Microbiology 3, no. 11 (2018): 1314–1326, 10.1038/s41564-018-0258-8. PMC6217937 30349080

[54] V. Raghavan , E. C. Lowe , G. E. Townsend 2nd , D. N. Bolam , and E. A. Groisman , “Tuning Transcription of Nutrient Utilization Genes to Catabolic Rate Promotes Growth in a Gut Bacterium,” Molecular Microbiology 93, no. 5 (2014): 1010–1025, 10.1111/mmi.12714. 25041429

[55] A. Cartmell , E. C. Lowe , A. Baslé , et al., “How Members of the Human Gut Microbiota Overcome the Sulfation Problem Posed by Glycosaminoglycans,” Proceedings of the National Academy of Sciences 114, no. 27 (2017): 7037–7042, 10.1073/pnas.1704367114. PMC5502631 28630303

[56] J. M. Holmen Larsson , K. A. Thomsson , A. M. Rodriguez‐Pineiro , H. Karlsson , and G. C. Hansson , “Studies of Mucus in Mouse Stomach, Small Intestine, and Colon. III. Gastrointestinal Muc5ac and Muc2 Mucin O‐Glycan Patterns Reveal a Regiospecific Distribution,” American Journal of Physiology: Gastrointestinal and Liver Physiology 305 (2013): G357–G363. 10.1152/ajpgi.00048.2013 PMC3761246 23832516

[57] G. Prezza , R. T. Fansler , T. Guest , et al., “An RNA Regulates Iron Homeostasis and Host Mucus Colonization in *Bacteroides thetaiotaomicron* ,” BioRxiv (2025): 2025.09.08.672848.

[58] L. Garcia‐Bayona and L. E. Comstock , “Streamlined Genetic Manipulation of Diverse Bacteroides and Parabacteroides Isolates From the Human Gut Microbiota,” MBio 10, no. 4 (2019): e01762–19, 10.1128/mBio.01762-19. PMC6692515 31409684

[59] N. E. Sanjana , O. Shalem , and F. Zhang , “Improved Vectors and Genome‐Wide Libraries for CRISPR Screening,” Nature Methods 11, no. 8 (2014): 783–784, 10.1038/nmeth.3047. PMC4486245 25075903

[60] M. Mimee , A. C. Tucker , C. A. Voigt , and T. K. Lu , “Programming a Human Commensal Bacterium, *Bacteroides thetaiotaomicron*, to Sense and Respond to Stimuli in the Murine Gut Microbiota,” Cell Systems 2, no. 3 (2016): 214, 10.1016/j.cels.2016.03.007. 27135367

[61] T. Wu , E. Hu , S. Xu , et al., “ClusterProfiler 4.0: A Universal Enrichment Tool for Interpreting Omics Data,” Innovation (Camb) 2 (2021): 100141. 10.1016/j.xinn.2021.100141 PMC8454663 34557778

[62] M. I. Love , W. Huber , and S. Anders , “Moderated Estimation of Fold Change and Dispersion for RNA‐Seq Data With DESeq2,” Genome Biology 15, no. 12 (2014): 550, 10.1186/s13059-014-0550-8. PMC4302049 25516281

[63] A. V. Sastry , Y. Gao , R. Szubin , et al., “The *Escherichia coli* Transcriptome Mostly Consists of Independently Regulated Modules,” Nature Communications 10, no. 1 (2019): 5536, 10.1038/s41467-019-13483-w. PMC6892915 31797920

