# Supplementary material for: Determination of Gene‐Level Fitness Contributions Across the Mucin‐Utilization Network of Bacteroides thetaiotaomicro n
Source: Adv Sci (Weinh). 2026 Oct 4:e78118. Online ahead of print. doi: 10.1002/advs.78118 (PMC13634575; doi:10.1002/advs.78118)
Supplement: Supplementary file 1 [file ADVS-9999-e78118-s012.pdf]

**Supplemental information**

# Determination of gene-level fitness contributions across the mucin-utilization network of *Bacteroides thetaiotaomicron*

Kangsan Kim[a,b,†], Jiwon Lee [a,b,†], Minjeong Kang[b,c], Sung Sun Yim[a,b,c], Donghui Choe[d,*], and Byung-Kwan Cho[a,b,c,*]

[a]Graduate School of Engineering Biology, Korea Advanced Institute of Science and Technology, Daejeon 34141, Republic of Korea.
[b]KAIST Institute for the BioInnovation, Korea Advanced Institute of Science and Technology, Daejeon 34141, Republic of Korea.
[c]Department of Biological Sciences, Korea Advanced Institute of Science and Technology, Daejeon 34141, Republic of Korea.
[d]Department of Biological Sciences, Sungkyunkwan University, Suwon 32267, Republic of Korea.
[†]These authors contributed equally to this work
*Correspondence: D.C (dchoe@skku.edu) and B.K-C. (bcho@kaist.ac.kr)

## Contents

**Supplemental Text**
**Text S1.** Condition specific fitness profile in genes associated with amino acid biosynthetic pathways
**Text S2.** MM-muc specific fitness defect in BT_0771
**Text S3.** iModulon-based view of condition-dependent transcriptional remodeling between MM-glc and MM-muc
**Text S4.** Experimental setting and quality control for in vivo CRISPRi screen
**Text S5.** Quadrant-level analysis of fitness changes between MM-muc and in vivo.

**Supplemental Tables (all uploaded as separate .xlsx files)**
**Table S1.** A synthetic sgRNA library targeting *Bacteroides thetaiotaomicron* VPI-5482 coding sequences
**Table S2.** Sequencing statistics of CRISPRi data
**Table S3.** Raw sgRNA read counts
**Table S4.** Genes with significantly changed z-scores in either MM-glc or MM-muc
**Table S5.** z-scores in select KEGG categories
**Table S6.** Gene expression profile of select iModulons associated with amino acid metabolism
**Table S7.** Genes with significantly changed z-scores *in vivo*
**Table S8**. Gene lists in the four quadrants in Fig. 7c.
**Table S9.** Fitness scores in MM-glc, MM-muc and colon across selected categories
**Table S10.** Plasmids and primers used in this study
**Table S11.** psgRNA vector with NT guide sequence

**Supplemental Figures**
**Figure S1.** Schematic of the genome-scale CRISPRi library construction *in B. thetaiotaomicron*
**Figure S2.** Quality assessment of the genome-scale CRISPRi sgRNA library and positional distribution of sgRNA-derived fitness scores
**Figure S3.** Benchmarking CRISPRi-derived fitness scores against previously defined essential genes and functional categories
**Figure S4.** Reproducibility of genome-wide CRISPRi fitness profiles across biological replicates
**Figure S5.** Growth phenotypes of *cps* mutants and cps4-repressed strains.
**Figure S6.** Fitness and transcriptional profiles of the eight capsular *cps* loci in MM-glc and MM-muc
**Figure S7.** Heatmap displaying genes predicted to encode other membrane-bound, glycan sensing and transport machineries.
**Figure S8.** Distribution of fitness z-scores for genes categorized by PULs statistically significant in MM-muc.
**Figure S9.** Genomic context, expression, and condition-dependent fitness of the *gibS*–BT_0769–BT_0771 locus
**Figure S10.** Comparison of iModulon activities between MM-glc and MM-muc

**Figure S11.** An overview of the modular changes in transcriptome expression between MM-glc and MM-muc
**Figure S12.** Comparison of growth phenotypes between NT and BT0623-repressed strains
**Figure S13.** Quality assessment and functional validation of the in vivo CRISPRi screen during murine colonization
**Figure S14.** Z-score correlation plot between MM-glc and in vivo (colon)
**Figure S15.** Category-level root mean square error (RMSE) comparison of in vitro fitness profiles against the in vivo colon profile

**Supplemental Text**

## Text S1. The three-tied mucin utilization network in *B. thetaiotaomicron*

Initial engagement of host-derived mucin glycans is likely shaped by the cell-surface architecture of *B. thetaiotaomicron*. Surface glycan-binding proteins, including SusD-like proteins, TonB-dependent transporters and CBM-containing enzymes, can promote substrate sensing, retention and access of mucin-derived glycan motifs at the outer membrane [1-3]. In parallel, capsular polysaccharides may indirectly influence mucin access by altering the steric properties of the cell surface [1] (**Figure. 1a**). Although direct evidence for CPS-mediated control of mucin access remains limited, the coordinated regulation of host-glycan-responsive PULs and capsule biosynthesis suggests that capsule remodeling is integrated with glycan-acquisition programs [4]. Following substrate encounter, mucin processing proceeds through coordinated activation of PULs, which couple glycan sensing and TonB-dependent transport with the activities of CAZymes (**Figure. 1b**) In particular, glycosidic hydrolases, sulfatases and mucin-associated peptidases that degrade mucin *O*-glycans into smaller oligo-, mono-saccharides and glycopeptides [5-8]. The resulting complex oligosaccharides are then captured and imported into the periplasm via TonB-dependent transporters, where a consortium of CAZymes further depolymerize them into transportable mono- and disaccharides. *Bacteroides* utilizes mucin-derived sugars liberated from mucin, where major constituents include fucose, galactose, *N*-acetylglucosamine (GlcNAc) and *N*-acetylgalactosamine (GalNAc) [9]. These liberated monosaccharides are routed through highly specific, dedicated catabolic pathways [3, 10], and *B. thetaiotaomicron* is predicted to encode each of this catabolic pathway (**Figure. 1c**). For instance, it encodes a fucose utilization operon that enable isomerization and phosphorylation of fucose before entering central metabolism [11, 12]. In addition, it also encodes the Leloir pathway, as well as genes associated with transport of hexosamines into glycolysis [13]. As the result, it is expected that *B. thetaiotaomicron* utilize these catabolic pathways funnel carbon into the central metabolic pathways.

## Text S1. Condition specific fitness profile in genes associated with amino acid biosynthetic pathways.

Gene-level analysis further revealed condition-specific fitness dependencies In MM-glc, genes associated with methionine biosynthesis (BT_0180) and those that channel the glycolytic intermediate 3-phosphoglycerate into *de novo* serine biosynthesis (BT_1152 and BT_1153) were more extensively depleted (**Table S5**). Conversely, in MM-muc, genes involved in L-homoserine biosynthesis and degradation (BT_2403, BT_2413, and BT_3636) showed stronger depletion in MM-muc. Together, these results highlight condition-specific amino acid dependencies in *B. thetaiotaomicron* ,which recapitulates the previous observation that the specific metabolic bottlenecks and pathway dependencies undergo significant shifts depending on the primary carbon source [14].

## Text S2. MM-muc specific fitness defect in BT_0771

Despite the broad functional complementation observed across most GHs, BT_0771 which encodes a GH13 glucan-branching enzyme, showed a notable fitness defect (z-score < -1.0) (**Figure. 5a**). Genomic context analysis revealed that BT_0771 resides within the BT_0769-0771 operon, positioned immediately downstream of the well-characterized small RNA, *gibS* (**Figure. S9a**). Consistent with our expression data, *gibS* is known to be induced by GlcNAc, mucin, or entry into the stationary phase, functioning as a repressor for specific downstream targets, including BT_0771 [15, 16]. Intriguingly, our CRISPRi results and previous transposon-insertion sequencing data [14] demonstrate that targeted knockdown or inactivation of BT_0771 leads to a fitness decline not only in MM-muc, but across a wide range of carbon-supplemented defined media (**Figure. S9b, c**). Together, these results point toward an intricate regulatory network. While the expression of BT_0771 is tightly repressed by *gibS* in the presence of glycans and their constituent sugars, maintaining a basal level of this enzyme appears to retain a non-trivial contribution to fitness across multiple physiological conditions.

**Text S3. iModulon-based view of condition-dependent transcriptional remodeling between MM-glc and MM-muc.**

To confirm whether such condition-specific gene essentiality translates to transcriptome-level changes, we referred to *B. thetaiotamoicron* iModulon framework [17]. iModulons are statistically inferred gene modules whose members covary across transcriptomes and therefore often reflect shared regulation or common physiological function [18, 19]. By projecting gene-level expression changes into module activities, iModulon analysis provides a systems-level view of how cellular programs are reallocated between conditions.

Differential iModulon activity analysis comparing MM-glc and MM-muc revealed a particularly strong remodeling among PUL-associated modules. Eight PUL iModulons was above the iModulon threshold of 3 in MM-muc, whereas only one PUL iModulon fell below the negative threshold (**Figure. S9**). Mapping these iModulons onto the pathway, activated PUL modules localized largely to the known polysaccharide-utilization genes (**Figure. S11**). Notably, all activated PUL iModulons except HTCS_Hya, HTCS_Man1_3, and SigM_PUL13_21 belonged to PULs induced by mucin *O*-glycans [5], indicating that the iModulon framework recapitulated the known mucin-responsive transcriptional program of *B. thetaiotaomicron* grown on mucin.

Amino acid metabolism represented a second major axis of transcriptional remodeling between MM-glc and MM-muc (**Figure. S10**). In particular, the Glu/Asp iModulon showed higher activity in MM-muc, whereas the Glu/Gln/Asn, Trp/Tyr, and Val/Leu/Ile iModulons showed lower activity in MM-muc than in MM-glc. Mapping these iModulons onto the pathway context by and large recapitulated the amino acid metabolic pathway (**Figure. S11**). At gene-level, those assigned to the Glu/Asp iModulon showed a significant, uniform up-regulation in MM-muc, whereas genes in the Glu/Gln/Asn iModulon the Trp/Tyr and Val/Leu/Ile iModulons (pathways) were uniformly downregulated (**Figure. S11**, **Table S6**). Together, these results indicate that the shift from glucose to mucin is accompanied by broad up-regulation of PULs, alongside with a selective redistribution of transcriptional changes across amino acid biosynthetic programs as consistent with the result in **Figure. 3b**. Together, these findings recapitulate previous observations that specific metabolic bottlenecks and

pathway dependencies undergo significant shifts depending on the primary carbon source [14].

**Text S4. Experimental setting and quality control for *in vivo* CRISPRi screen.**

Mice were treated with antibiotics for clearance of native gut microflora prior to library inoculation. CRISPRi library was allowed to propagate for 3 days before being sacrificed for NGS run (**Figure. 7A**). Read depths of colon samples were comparable to those of *in vitro* (**Table S2**). PCA analysis showed a high degree of similarity between biological replicates (n=7), with the exception of replicate #3 and #4, which separated from the main cluster, and exhibited a lower correlation between replicates (**Figure. S13a**, **b**). Consistent with this, sgRNA insertion frequency in replicates #3 and #4 displayed perturbed distribution patterns suggestive of over-depletion or technical errors and were omitted from downstream analysis (**Figure. S13c**). The z-score distribution showed a clear depletion on essential gene sets than the rest (**Figure. S13d**) [14], where strong negative fitness effects were concentrated in pathways associated with core growth functions, including translation, ribosome biogenesis, transcription, and cell envelope biogenesis (**Figure. S13e**), supporting the technical and biological fidelity of the colon CRISPRi screen.

**Text S5. Quadrant-level analysis of fitness changes between MM-muc and in vivo.**

We examined functional enrichments across the four quadrants of the fitness scatter plot (**Figure. 7c**; **Table S8**). Genes depleted in both MM-muc and the colon (Q3) were enriched for translation- and ribosome-associated functions, consistent with shared core growth requirements across the two environments. In contrast, genes displaying discordant fitness profiles between the two environments in Q2 were enriched for glycosyltransferase activity [GO:0016757], which include BT_3366, BT_3367, and BT_3368. Notably, their *in vivo* z-score distribution mirrored that of the MM-glc condition rather than the MM-muc (**Figure. 7c**, **Table S4**). This suggests that the observed discordance is driven by a mucin-specific fitness requirement rather than a general determinant of colon fitness. Although Q1 and Q4 lacked functional category enrichments, locus-specific analysis revealed distinct phenotypic patterns (**Table S8**). In Q1, highly dispensable gene sets tend to be PULs (or corresponding CAZys) and CPSs, in colon and MM-muc, respectively, highlighting their functional divergence between the two conditions. Q4 on the other hand, comprised a functionally heterogeneous set of genes with divergent fitness effects between the two conditions, implications of which remain unclear.

## Supplemental Figures

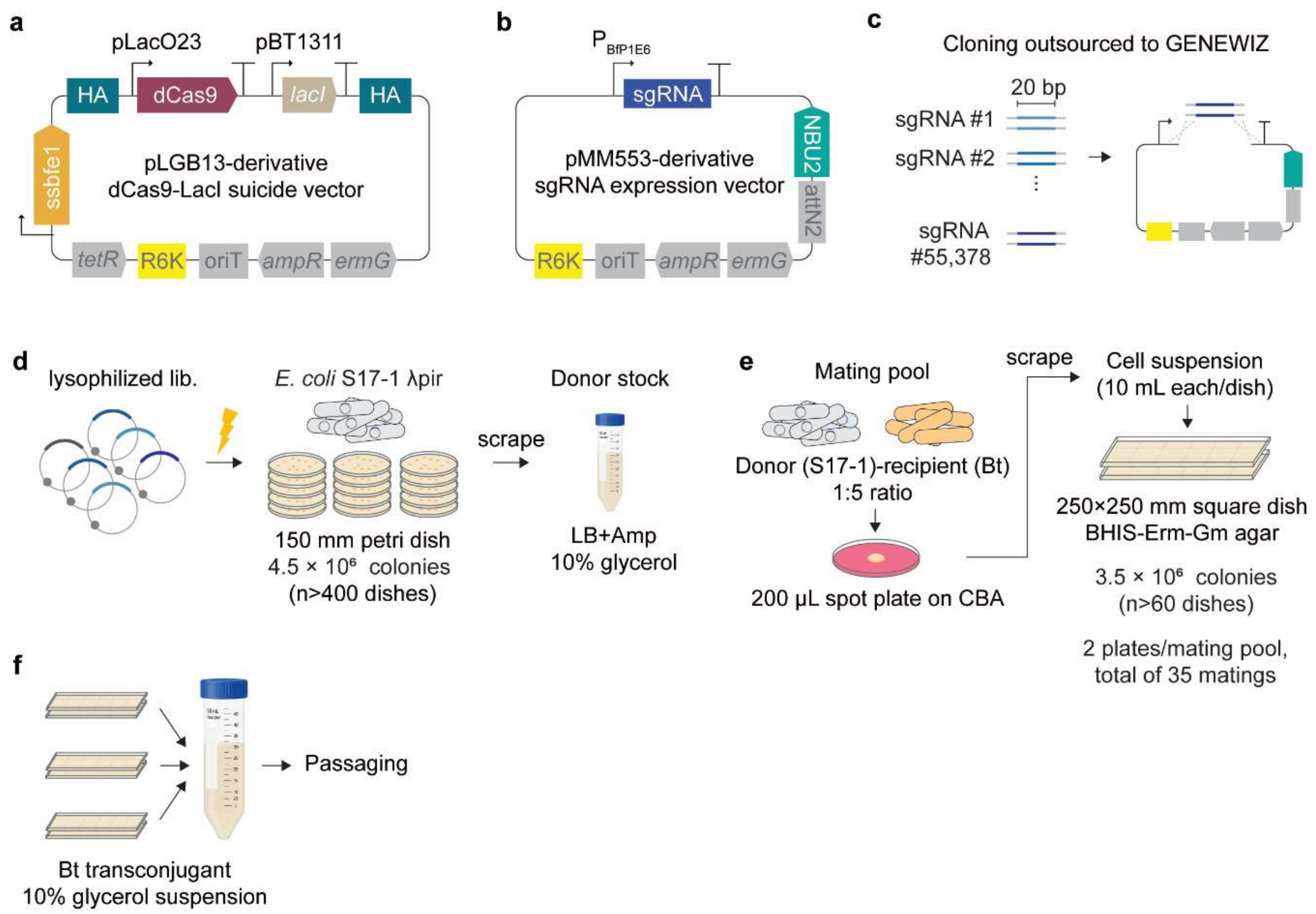


**Figure S1. Schematic of the genome-scale CRISPRi library construction in *B. thetaiotaomicron*.** **(a)** Schematic of the pLGB13-derived dCas9–LacI suicide vector used to introduce the inducible CRISPRi machinery into the *B. thetaiotaomicron* chromosome. **(b)** Schematic of the NBU2-containing, pMM553-derived sgRNA expression vector, in which sgRNA expression is driven by the constitutive P_BfP1E6 promoter. **(c)** Construction of the pooled sgRNA plasmid library. A total of 55,378 sgRNAs containing 20-bp gene-targeting sequences were cloned into the sgRNA expression vector. Vector library cloning was performed by GENEWIZ. **(d)** Generation of the *E. coli* S17-1 λpir donor library. The lyophilized plasmid library was transformed into S17-1 λpir and plated on 150-mm LB–ampicillin agar plates, yielding approximately $4.5 \times 10^6$ colonies across >400 plates. Colonies were pooled by scraping and preserved in LB containing ampicillin and 10% glycerol to generate the donor stock. **(e)** Transfer of the pooled sgRNA library into *B. thetaiotaomicron* by conjugation. Donor S17-1 λpir and recipient *B. thetaiotaomicron* cells were combined at a 1:5 donor-to-recipient ratio, and 200 µl of each mating mixture was spotted onto CBA plates. Following mating, cells were recovered by scraping, resuspended in 20 mL of BHIS, and plated (10 mL) onto 250 × 250-mm square dish containing BHIS agar with erythromycin and gentamicin for transconjugant selection. Two selection plates were used per mating pool across 35 independent matings, yielding approximately $3.5 \times 10^6$ transconjugant colonies from >60 plates. **(f)** Selected *B. thetaiotaomicron* transconjugants were pooled, preserved as 10% glycerol suspensions, and used for subsequent passaging and CRISPRi fitness screening.

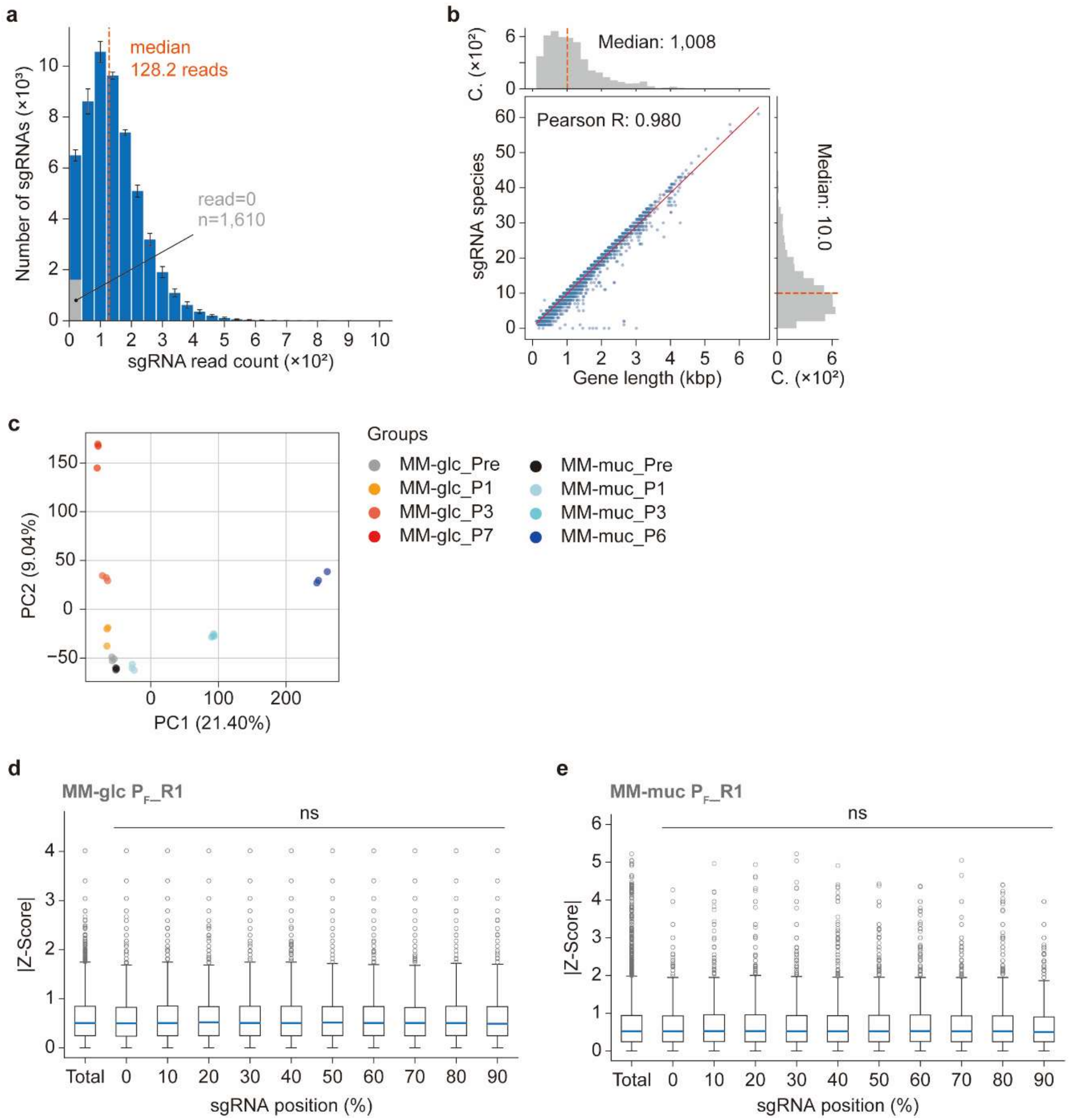


**Figure S2. Quality assessment of the genome-scale CRISPRi sgRNA library and positional distribution of sgRNA-derived fitness scores. (a)** Distribution of sequencing read counts for individual sgRNAs in the initial CRISPRi library. The orange dashed line indicates the median read count (128.2 reads per sgRNA). sgRNAs with zero reads are indicated in gray. Error bars represent variation across replicate measurements. **(b)** Correlation between gene length and the number of uniquely mapped sgRNAs per coding sequence. The central scatter plot shows a strong linear correlation between gene length and sgRNA count. Histograms show the distributions of CDS length and sgRNA count per gene; orange dashed lines indicate the respective medians. **(c)** Principal component analysis (PCA) of genome-wide sgRNA abundance profiles across the initial library and sequential passages in minimal medium containing glucose (MM-glc) or mucin (MM-muc). **(d, e)** Distribution of

sgRNA-derived z-scores as a function of target position along coding sequences in the final passage of MM-glc **(d)** and MM-muc **(e)** for representative replicate Box plots show the median and interquartile range, with whiskers extending to 1.5× the interquartile range. ns: non-significant.

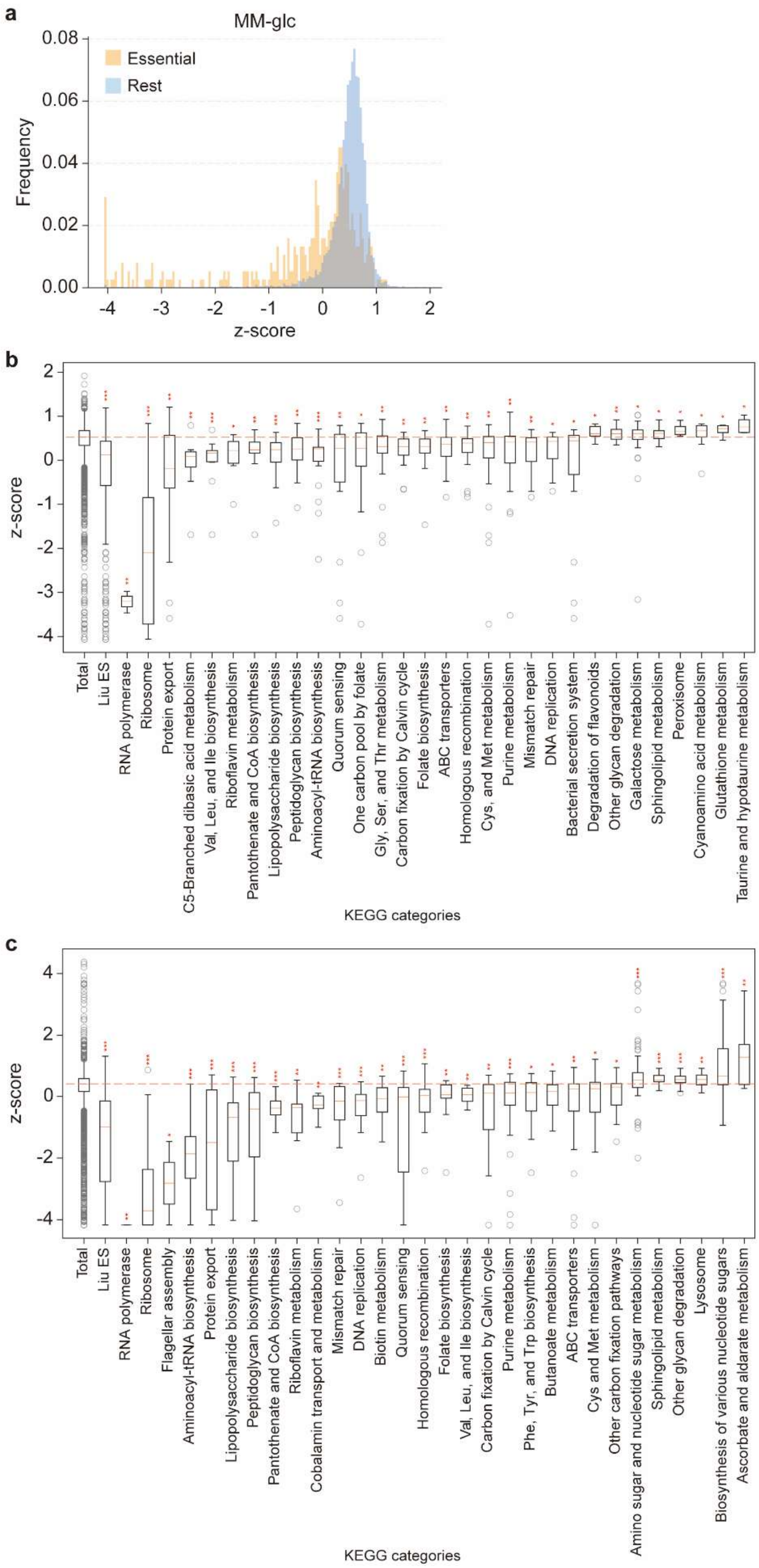

a
MM-glc
Essential
Rest
Frequency
0.08
0.06
0.04
0.02
0.00
-4
-3
-2
-1
0
1
2
z-score
b
z-score
2
1
0
-1
-2
-3
-4
Total
Liu ES
RNA polymerase
Ribosome
Protein export
C5-Branched dibasic acid metabolism
Val, Leu, and Ile biosynthesis
Riboflavin metabolism
Pantothenate and CoA biosynthesis
Lipopolysaccharide biosynthesis
Peptidoglycan biosynthesis
Aminoacyl-tRNA biosynthesis
Quorum sensing
One carbon pool by folate
Gly, Ser, and Thr metabolism
Carbon fixation by Calvin cycle
Folate biosynthesis
ABC transporters
Homologous recombination
Cys, and Met metabolism
Purine metabolism
Mismatch repair
DNA replication
Bacterial secretion system
Degradation of flavonoids
Other glycan degradation
Galactose metabolism
Sphingolipid metabolism
Peroxisome
Cyanoamino acid metabolism
Glutathione metabolism
Taurine and hypotaurine metabolism
KEGG categories
c
z-score
4
2
0
-2
-4
Total
Liu ES
RNA polymerase
Ribosome
Flagellar assembly
Aminoacyl-tRNA biosynthesis
Protein export
Lipopolysaccharide biosynthesis
Peptidoglycan biosynthesis
Pantothenate and CoA biosynthesis
Riboflavin metabolism
Cobalamin transport and metabolism
Mismatch repair
DNA replication
Biotin metabolism
Quorum sensing
Homologous recombination
Folate biosynthesis
Val, Leu, and Ile biosynthesis
Carbon fixation by Calvin cycle
Purine metabolism
Phe, Tyr, and Trp biosynthesis
Butanoate metabolism
ABC transporters
Cys and Met metabolism
Other carbon fixation pathways
Amino sugar and nucleotide sugar metabolism
Sphingolipid metabolism
Other glycan degradation
Lysosome
Biosynthesis of various nucleotide sugars
Ascorbate and aldarate metabolism
KEGG categories

**Figure S3. Benchmarking CRISPRi-derived fitness scores against previously defined essential genes and functional categories. (a)** Distribution of gene-level fitness z-scores for genes previously classified as essential versus the remainder of the genome in MM-glc. (b,c) Box plots showing the distribution of gene-level z-scores across selected KEGG functional categories in **(b)** MM-glc and **(c)** MM-muc. The category labeled Liu ES denotes the set of essential genes defined previously [14].

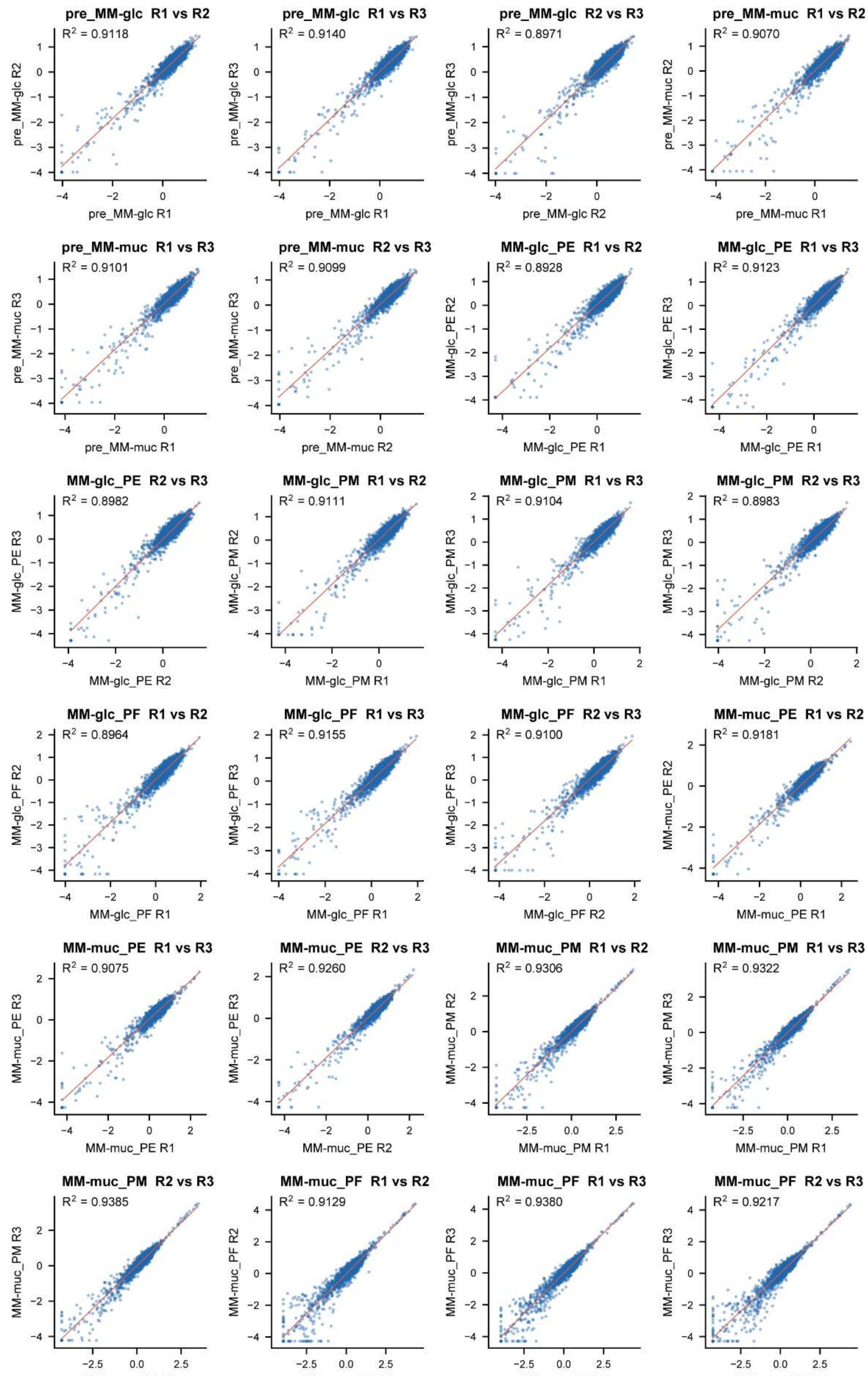
pre_MM-glc R1 vs R2
R2 = 0.9118
pre_MM-glc R2
pre_MM-glc R1
pre_MM-glc R1 vs R3
R2 = 0.9140
pre_MM-glc R3
pre_MM-glc R1
pre_MM-glc R2 vs R3
R2 = 0.8971
pre_MM-glc R3
pre_MM-glc R2
pre_MM-muc R1 vs R2
R2 = 0.9070
pre_MM-muc R2
pre_MM-muc R1
pre_MM-muc R1 vs R3
R2 = 0.9101
pre_MM-muc R3
pre_MM-muc R1
pre_MM-muc R2 vs R3
R2 = 0.9099
pre_MM-muc R3
pre_MM-muc R2
MM-glc_PE R1 vs R2
R2 = 0.8928
MM-glc_PE R2
MM-glc_PE R1
MM-glc_PE R1 vs R3
R2 = 0.9123
MM-glc_PE R3
MM-glc_PE R1
MM-glc_PE R2 vs R3
R2 = 0.8982
MM-glc_PE R3
MM-glc_PE R2
MM-glc_PM R1 vs R2
R2 = 0.9111
MM-glc_PM R2
MM-glc_PM R1
MM-glc_PM R1 vs R3
R2 = 0.9104
MM-glc_PM R3
MM-glc_PM R1
MM-glc_PM R2 vs R3
R2 = 0.8983
MM-glc_PM R3
MM-glc_PM R2
MM-glc_PF R1 vs R2
R2 = 0.8964
MM-glc_PF R2
MM-glc_PF R1
MM-glc_PF R1 vs R3
R2 = 0.9155
MM-glc_PF R3
MM-glc_PF R1
MM-glc_PF R2 vs R3
R2 = 0.9100
MM-glc_PF R3
MM-glc_PF R2
MM-muc_PE R1 vs R2
R2 = 0.9181
MM-muc_PE R2
MM-muc_PE R1
MM-muc_PE R1 vs R3
R2 = 0.9075
MM-muc_PE R3
MM-muc_PE R1
MM-muc_PE R2 vs R3
R2 = 0.9260
MM-muc_PE R3
MM-muc_PE R2
MM-muc_PM R1 vs R2
R2 = 0.9306
MM-muc_PM R2
MM-muc_PM R1
MM-muc_PM R1 vs R3
R2 = 0.9322
MM-muc_PM R3
MM-muc_PM R1
MM-muc_PM R2 vs R3
R2 = 0.9385
MM-muc_PM R3
MM-muc_PM R2
MM-muc_PF R1 vs R2
R2 = 0.9129
MM-muc_PF R2
MM-muc_PF R1
MM-muc_PF R1 vs R3
R2 = 0.9380
MM-muc_PF R3
MM-muc_PF R1
MM-muc_PF R2 vs R3
R2 = 0.9217
MM-muc_PF R3
MM-muc_PF R2

**Figure S4. Reproducibility of genome-wide CRISPRi fitness profiles across biological replicates.** Pairwise scatter plots comparing gene-level fitness z-scores between biological replicates for the initial library (pre) and for sequential passages in MM-glc or MM-muc.

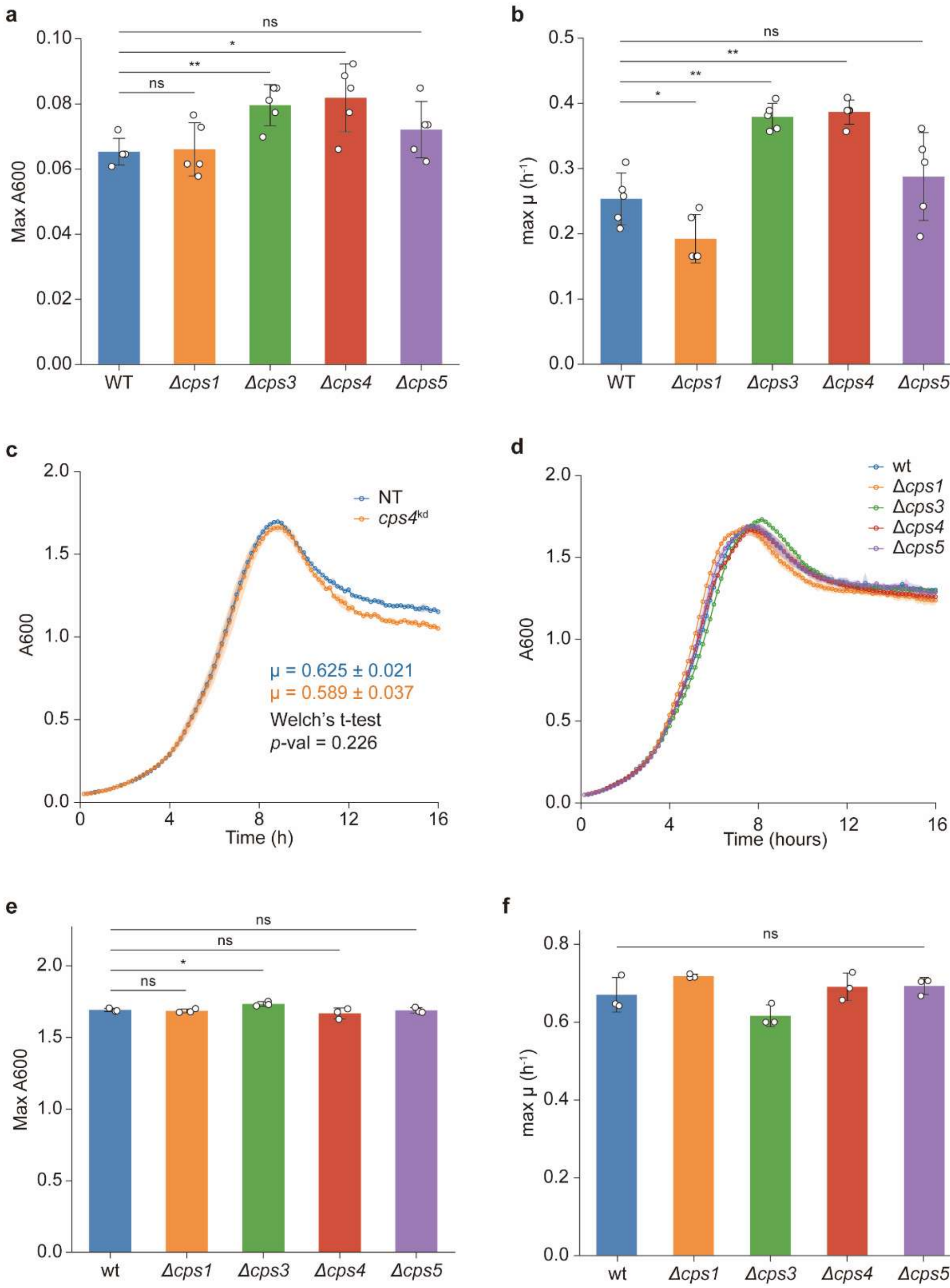


**Figure S5. Growth phenotypes of *cps* mutants and cps4-repressed strains.** . Measured growth parameters of **(a)** maximum growth rates and **(b)** specific growth rates between WT and Δ*cps* strains in MM-muc. **(c)** Growth kinetics of NT control versus *cps4*$^{kd}$ strain in MM-glc. **(d)** Growth kinetics of WT versus four *cps* deletion strains. Measured growth parameters of **(e)** maximum growth rates and **(f)** specific growth rates between WT and Δ*cps* strains. Significance was determined by Welch's t-test. Asterisk represents statistical significance * *p*-val < 0.05; ** *p*-val < 0.01.

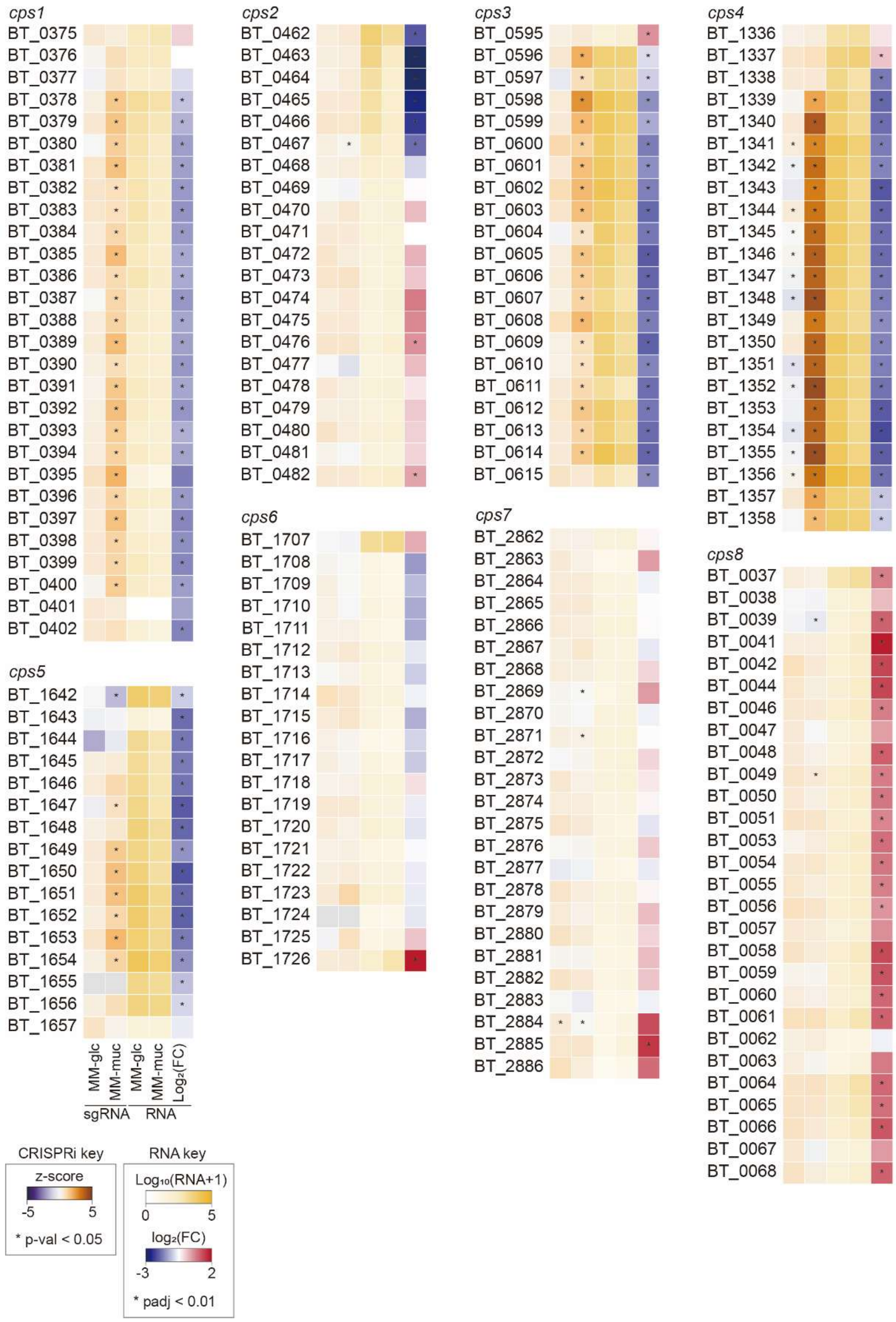


**Figure S6. Fitness and transcriptional profiles of the eight capsular *cps* loci in MM-glc and MM-muc.** Heatmaps showing gene-level CRISPRi fitness scores and transcript abundance for all annotated genes within the eight capsular polysaccharide (*cps1*– *cps8*) loci

of *B. thetaiotaomicron*. For each locus, the first two columns represent CRISPRi-derived fitness z-scores in MM-glc and MM-muc, respectively, and the next three columns represent RNA expression in MM-glc, MM-muc, and the corresponding $\log_2$ fold change ($\log_2$(FC)) between the two conditions. Asterisks denote statistical significance ($p < 0.05$ for CRISPRi; adjusted $p < 0.01$ for RNA-seq).

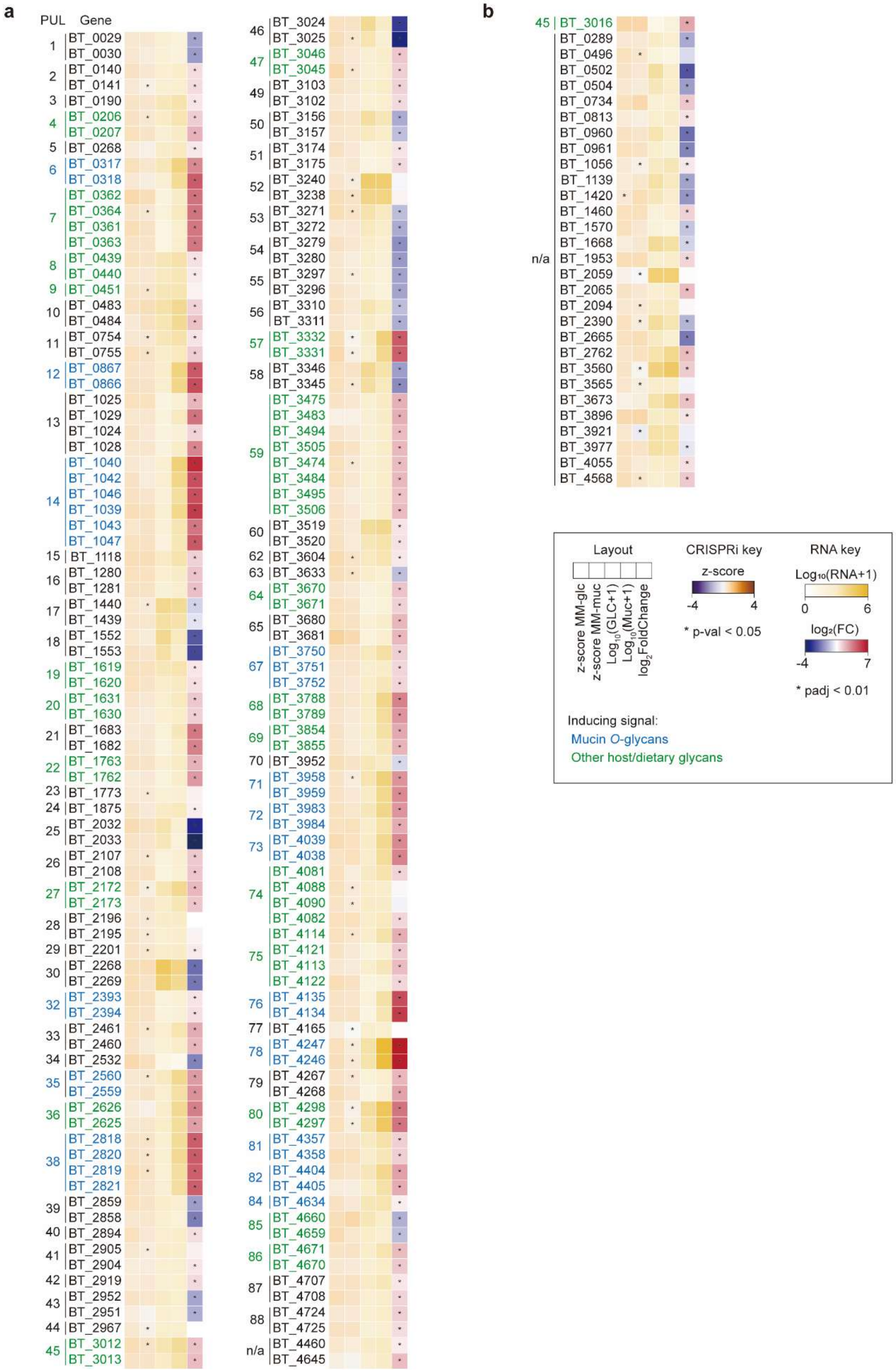
a
PUL Gene
1 BT_0029 BT_0030
2 BT_0140 BT_0141
3 BT_0190
4 BT_0206 BT_0207
5 BT_0268
6 BT_0317 BT_0318
7 BT_0362 BT_0364 BT_0361 BT_0363
8 BT_0439 BT_0440
9 BT_0451
10 BT_0483 BT_0484
11 BT_0754 BT_0755
12 BT_0867 BT_0866
13 BT_1025 BT_1029 BT_1024 BT_1028
14 BT_1040 BT_1042 BT_1046 BT_1039 BT_1043 BT_1047
15 BT_1118
16 BT_1280 BT_1281
17 BT_1440 BT_1439
18 BT_1552 BT_1553
19 BT_1619 BT_1620
20 BT_1631 BT_1630
21 BT_1683 BT_1682
22 BT_1763 BT_1762
23 BT_1773
24 BT_1875
25 BT_2032 BT_2033
26 BT_2107 BT_2108
27 BT_2172 BT_2173
28 BT_2196 BT_2195
29 BT_2201
30 BT_2268 BT_2269
32 BT_2393 BT_2394
33 BT_2461 BT_2460
34 BT_2532
35 BT_2560 BT_2559
36 BT_2626 BT_2625
38 BT_2818 BT_2820 BT_2819 BT_2821
39 BT_2859 BT_2858
40 BT_2894
41 BT_2905 BT_2904
42 BT_2919
43 BT_2952 BT_2951
44 BT_2967
45 BT_3012 BT_3013
46 BT_3024 BT_3025
47 BT_3046 BT_3045
49 BT_3103 BT_3102
50 BT_3156 BT_3157
51 BT_3174 BT_3175
52 BT_3240 BT_3238
53 BT_3271 BT_3272
54 BT_3279 BT_3280
55 BT_3297 BT_3296
56 BT_3310 BT_3311
57 BT_3332 BT_3331
58 BT_3346 BT_3345
59 BT_3475 BT_3483 BT_3494 BT_3505 BT_3474 BT_3484 BT_3495 BT_3506
60 BT_3519 BT_3520
62 BT_3604
63 BT_3633
64 BT_3670 BT_3671
65 BT_3680 BT_3681
67 BT_3750 BT_3751 BT_3752
68 BT_3788 BT_3789
69 BT_3854 BT_3855
70 BT_3952
71 BT_3958 BT_3959
72 BT_3983 BT_3984
73 BT_4039 BT_4038
74 BT_4081 BT_4088 BT_4090 BT_4082
75 BT_4114 BT_4121 BT_4113 BT_4122
76 BT_4135 BT_4134
77 BT_4165
78 BT_4247 BT_4246
79 BT_4267 BT_4268
80 BT_4298 BT_4297
81 BT_4357 BT_4358
82 BT_4404 BT_4405
84 BT_4634
85 BT_4660 BT_4659
86 BT_4671 BT_4670
87 BT_4707 BT_4708
88 BT_4724 BT_4725
n/a BT_4460 BT_4645
b
45 BT_3016
n/a BT_0289 BT_0496 BT_0502 BT_0504 BT_0734 BT_0813 BT_0960 BT_0961 BT_1056 BT_1139 BT_1420 BT_1460 BT_1570 BT_1668 BT_1953 BT_2059 BT_2065 BT_2094 BT_2390 BT_2665 BT_2762 BT_3560 BT_3565 BT_3673 BT_3896 BT_3921 BT_3977 BT_4055 BT_4568
Layout
z-score MM-glc
z-score MM-muc
Log10(GLC+1)
Log10(Muc+1)
log2FoldChange
CRISPRi key
z-score
-4 4
* p-val < 0.05
RNA key
Log10(RNA+1)
0 6
log2(FC)
-4 7
* padj < 0.01
Inducing signal:
Mucin O-glycans
Other host/dietary glycans

**Figure S7. Heatmap displaying genes predicted to encode other membrane-bound, glycan sensing and transport machineries. (a)** SusCD and **(b)** TonB-dependent transport systems. Genes significantly changed in terms of either fitness (z-score with *p*-val < 0.05; Mann-Whitney U) or expression (DEGs with *padj* < 0.01) are displayed. Asterisks (*) indicates statistical significance.

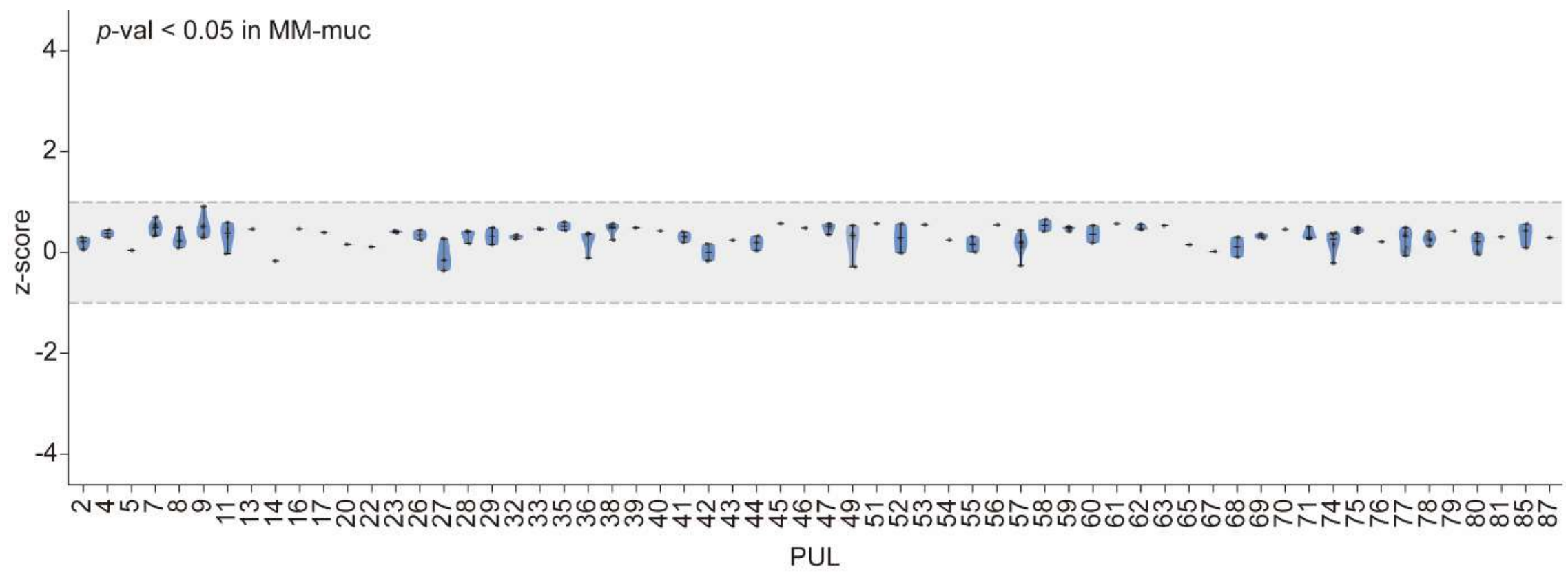


**Figure S8. Distribution of fitness z-scores for genes categorized by PULs statistically significant in MM-muc.** Violin plots show the spread of z-scores for individual genes within each family. Genes with |z-score| > 1.0 are highlighted.

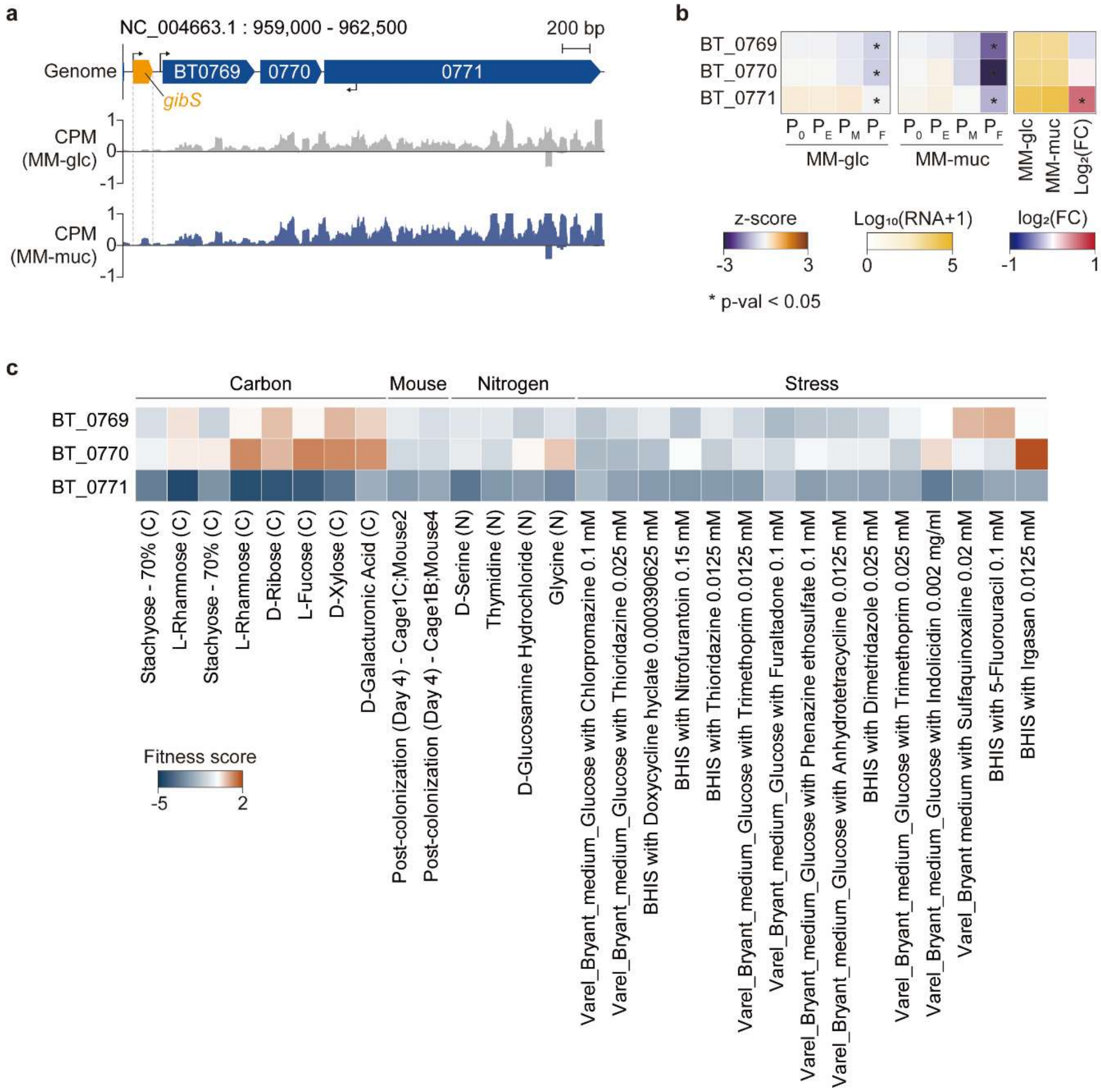


**Figure S9. Genomic context, expression, and condition-dependent fitness of the *gibS*–BT_0769–BT_0771 locus. (a)**, Genomic organization of the *gibS* locus and adjacent genes BT_0769, BT_0770, and BT_0771 in *B. thetaiotaomicron*. RNA-seq coverage profiles, shown as counts per million (CPM), are displayed for cells grown in MM-glc or MM-muc. **(b)** Heatmap showing CRISPRi-derived fitness z-scores for BT_0769, BT_0770, and BT_0771 across sequential passages in MM-glc and MM-muc, together with corresponding RNA abundance and differential expression between conditions. Asterisks indicate statistically significant values ($p < 0.05$). **(c)** Heatmap of fitness scores for BT_0769, BT_0770, and BT_0771 across diverse environmental conditions, grouped by carbon source, mouse colonization, nitrogen source, and stress-related perturbations.

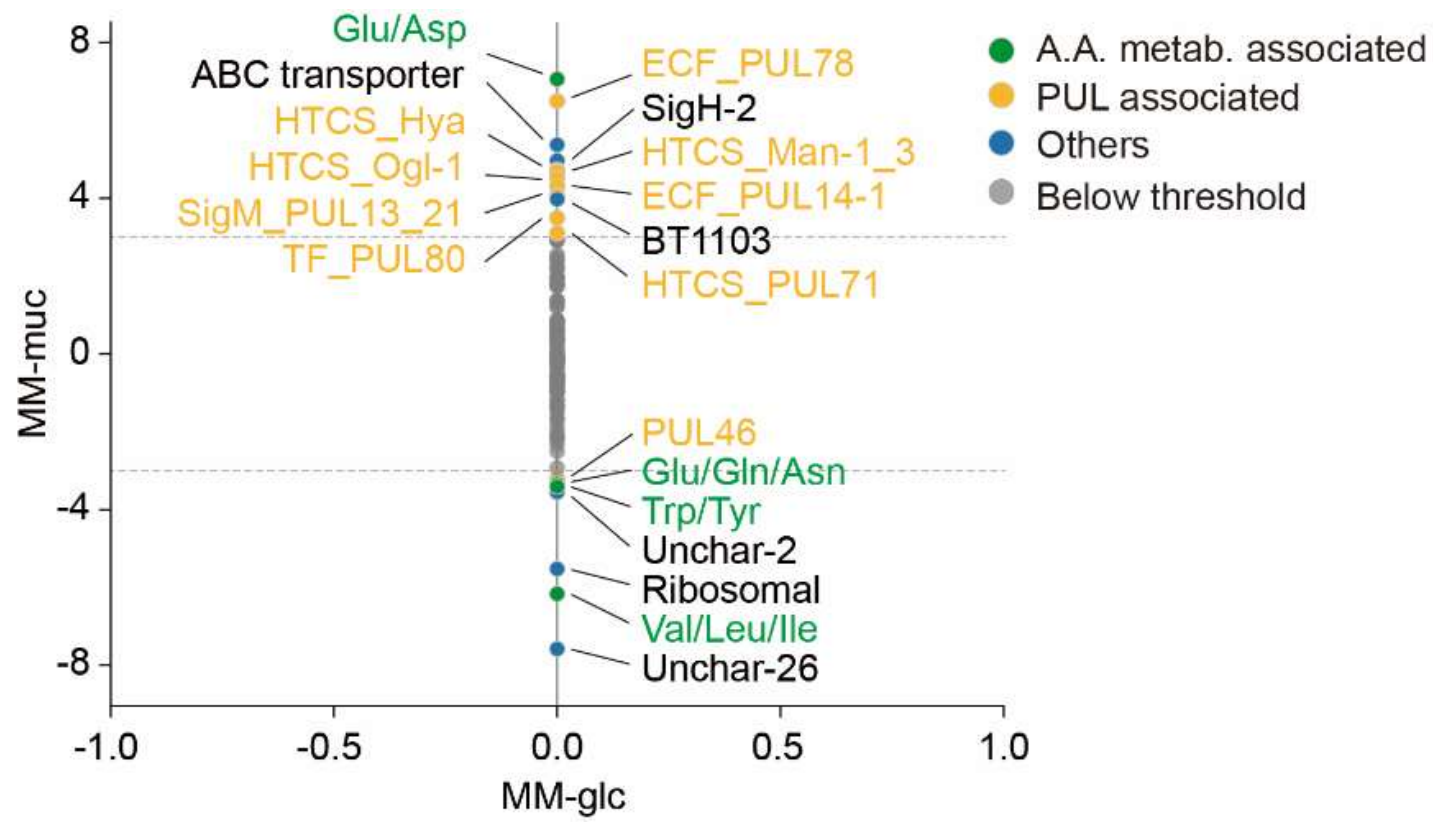


**Figure S10. Comparison of iModulon activities between MM-glc and MM-muc.** Dotted lines indicate activity thresholds (3 and -3, respectively). iModulons with distinct functional categories are highlighted in different colors. Abbreviations: A.A. metab. – amino acid metabolism; PUL: polysaccharide utilization loci.

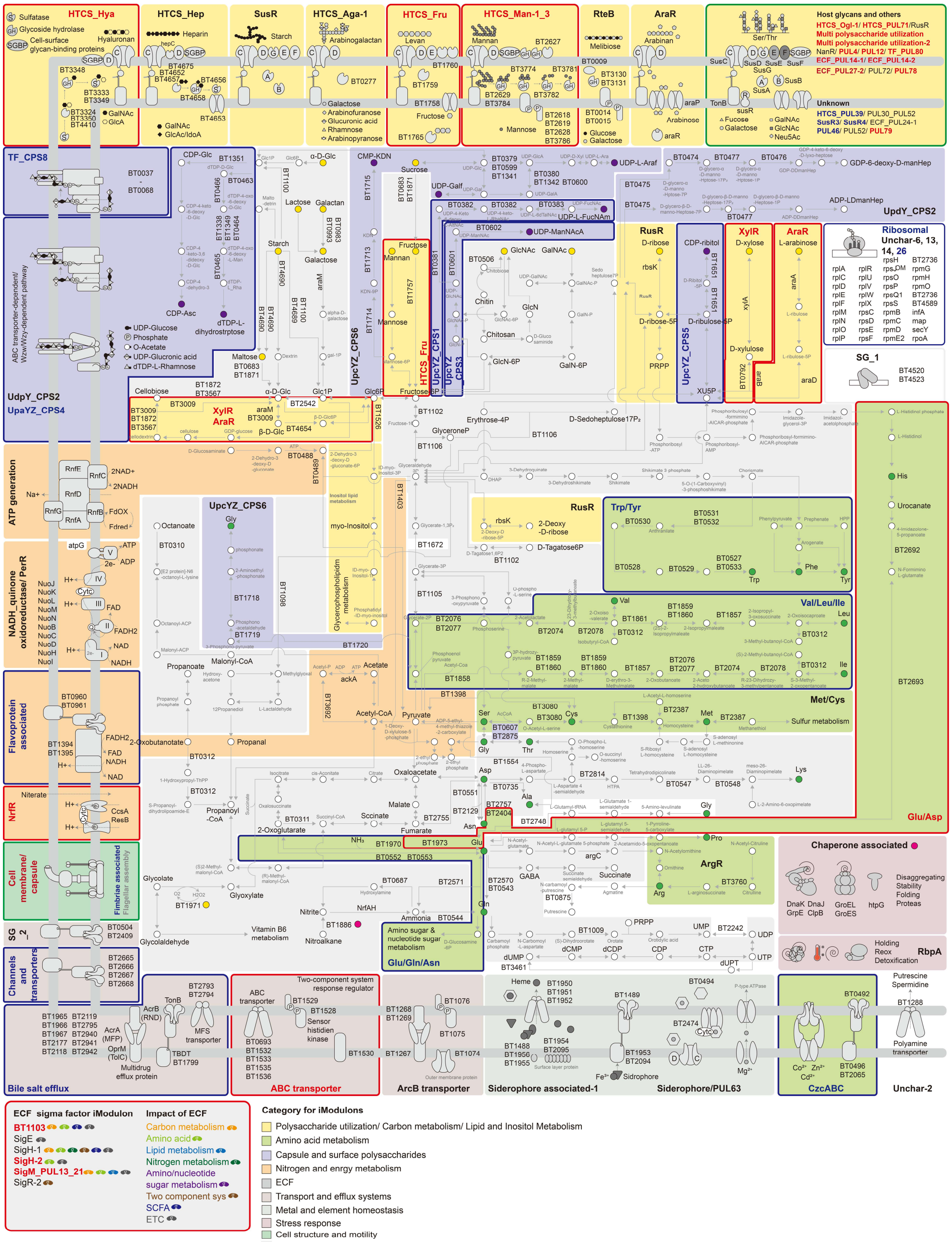


**Figure S11. An overview of the modular changes in transcriptome expression between MM-glc and MM-muc.** Global transcriptomic changes are visualized in the context of a curated genome-scale metabolic and functional map, highlighting condition-dependent

regulation of key cellular modules. iModulons with distinct functional categories are highlighted in unique color codes. Those with thick red or blue borders indicate iModulons with relatively higher (iModulon activity > 1) or lower activity (iModulon activity < -1) respectively in MM-muc than in MM-glc. For multi-iModulon boxes with contrasting iModulon activity (containing iModulons with both higher or lower iModulon activity) were highlighted in green.

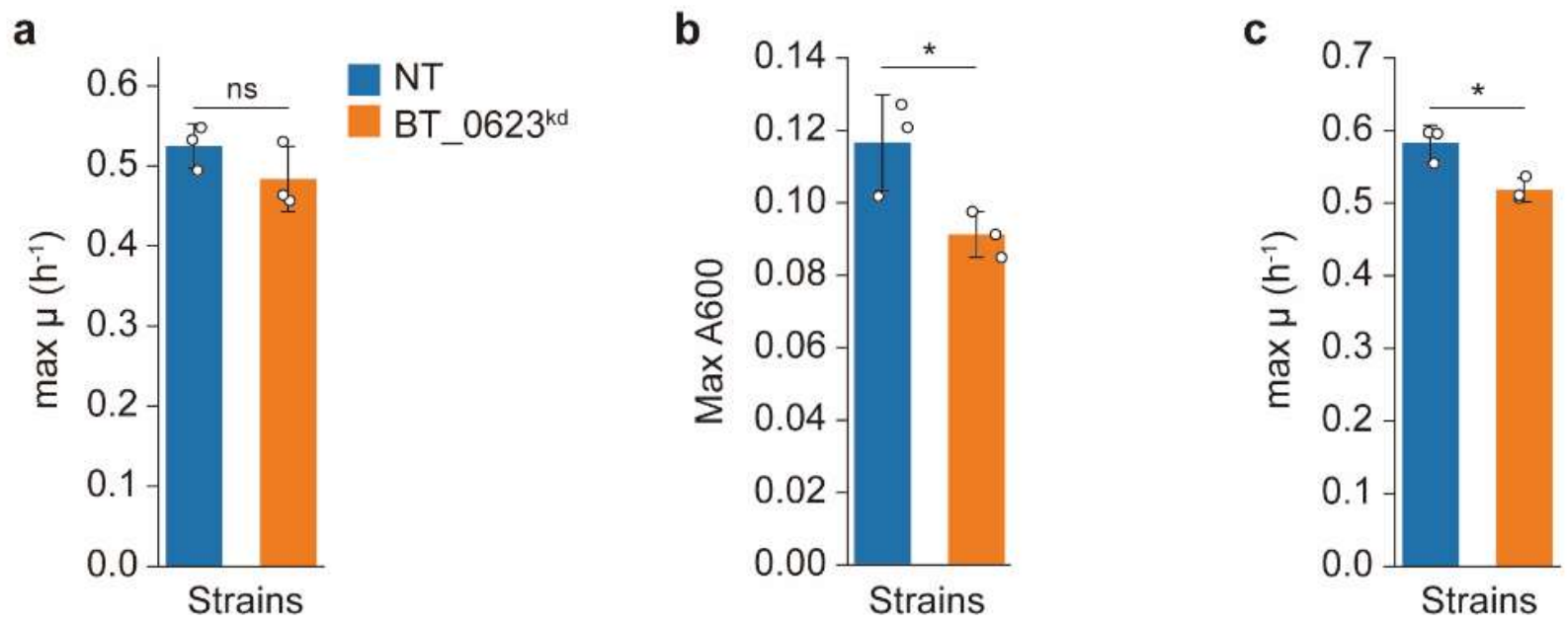


**Figure S12. Comparison of growth phenotypes between NT and BT0623-repressed strains. (a)** Specific growth rates in MM-glc. **(b)** maximum cell density in MM-muc. **(c)** Specific growth rates in MM-gal.

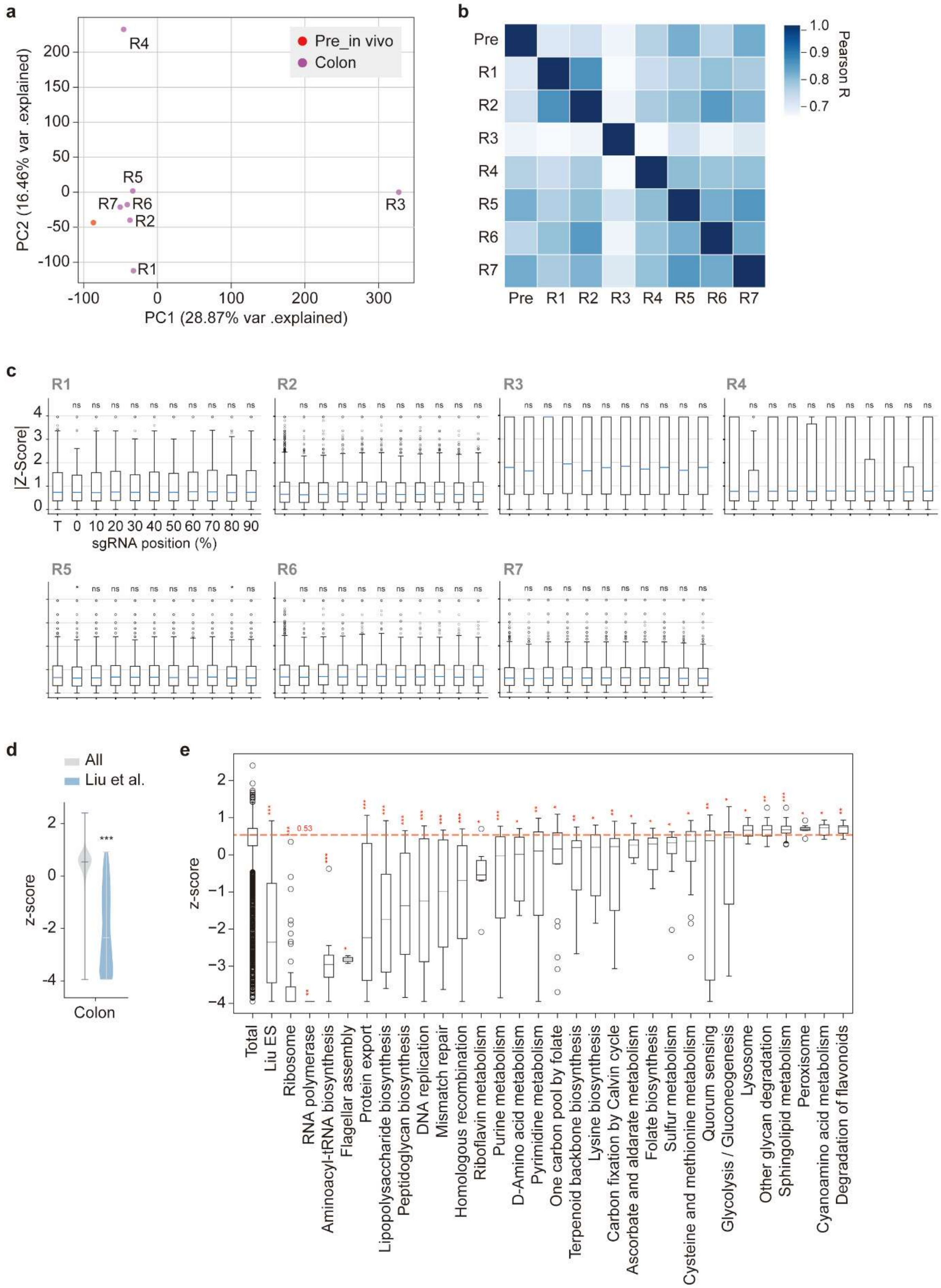
a
Pre_in vivo
Colon
R1
R2
R3
R4
R5
R6
R7
PC1 (28.87% var .explained)
PC2 (16.46% var .explained)
b
Pre
Pearson R
c
|Z-Score|
T 0 10 20 30 40 50 60 70 80 90
sgRNA position (%)
ns
d
All
Liu et al.
z-score
Colon
***
e
z-score
0.53
Total
Liu ES
Ribosome
RNA polymerase
Aminoacyl-tRNA biosynthesis
Flagellar assembly
Protein export
Lipopolysaccharide biosynthesis
Peptidoglycan biosynthesis
DNA replication
Mismatch repair
Homologous recombination
Riboflavin metabolism
Purine metabolism
D-Amino acid metabolism
Pyrimidine metabolism
One carbon pool by folate
Terpenoid backbone biosynthesis
Lysine biosynthesis
Carbon fixation by Calvin cycle
Ascorbate and aldarate metabolism
Folate biosynthesis
Sulfur metabolism
Cysteine and methionine metabolism
Quorum sensing
Glycolysis / Gluconeogenesis
Lysosome
Other glycan degradation
Sphingolipid metabolism
Peroxisome
Cyanoamino acid metabolism
Degradation of flavonoids

**Figure S13. Quality assessment and functional validation of the in vivo CRISPRi screen during murine colonization. (a)** PCA of genome-wide sgRNA abundance profiles comparing the input library (Pre_in vivo) with biological replicates recovered from the murine colon (R1–R7). **(b)** Heatmap showing pairwise Pearson correlation coefficients of genome-wide sgRNA abundance profiles among the input library and colon replicates. **(c)** Distribution of sgRNA-derived absolute z-scores as a function of relative target position along CDS for each colon replicate. Box plots show the median and interquartile range, with whiskers extending to 1.5× the interquartile range. ns: non-significant. **(d)** Violin plots comparing the distribution of gene-level fitness z-scores for all genes versus the previously defined essential-gene [14]. (***, $p < 0.001$). **(e)** Box plots showing the distribution of gene-level z-scores across selected KEGG functional categories in the colon CRISPRi screen.

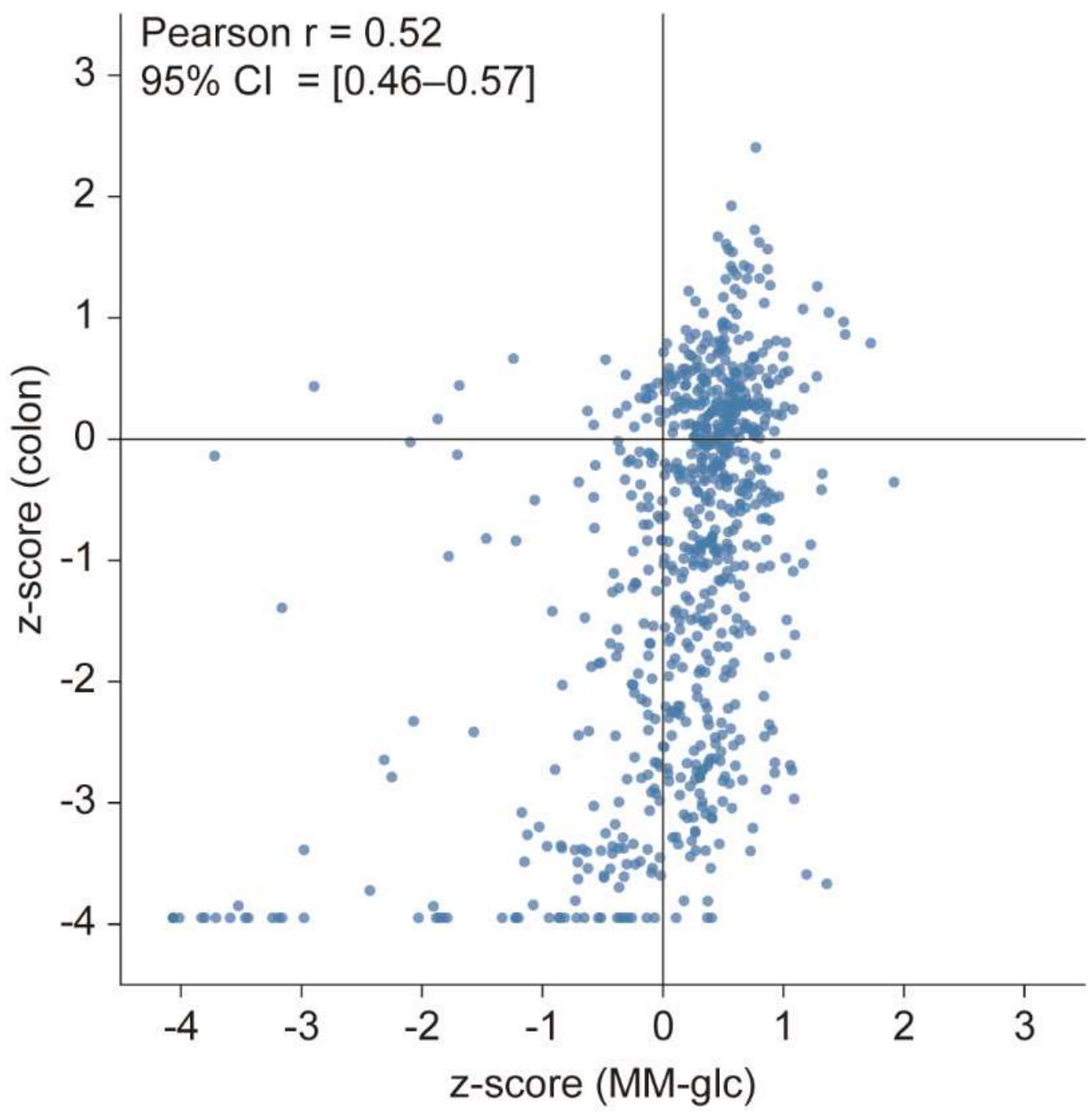


**Figure S14. Z-score correlation plot between MM-glc and in vivo (colon).**

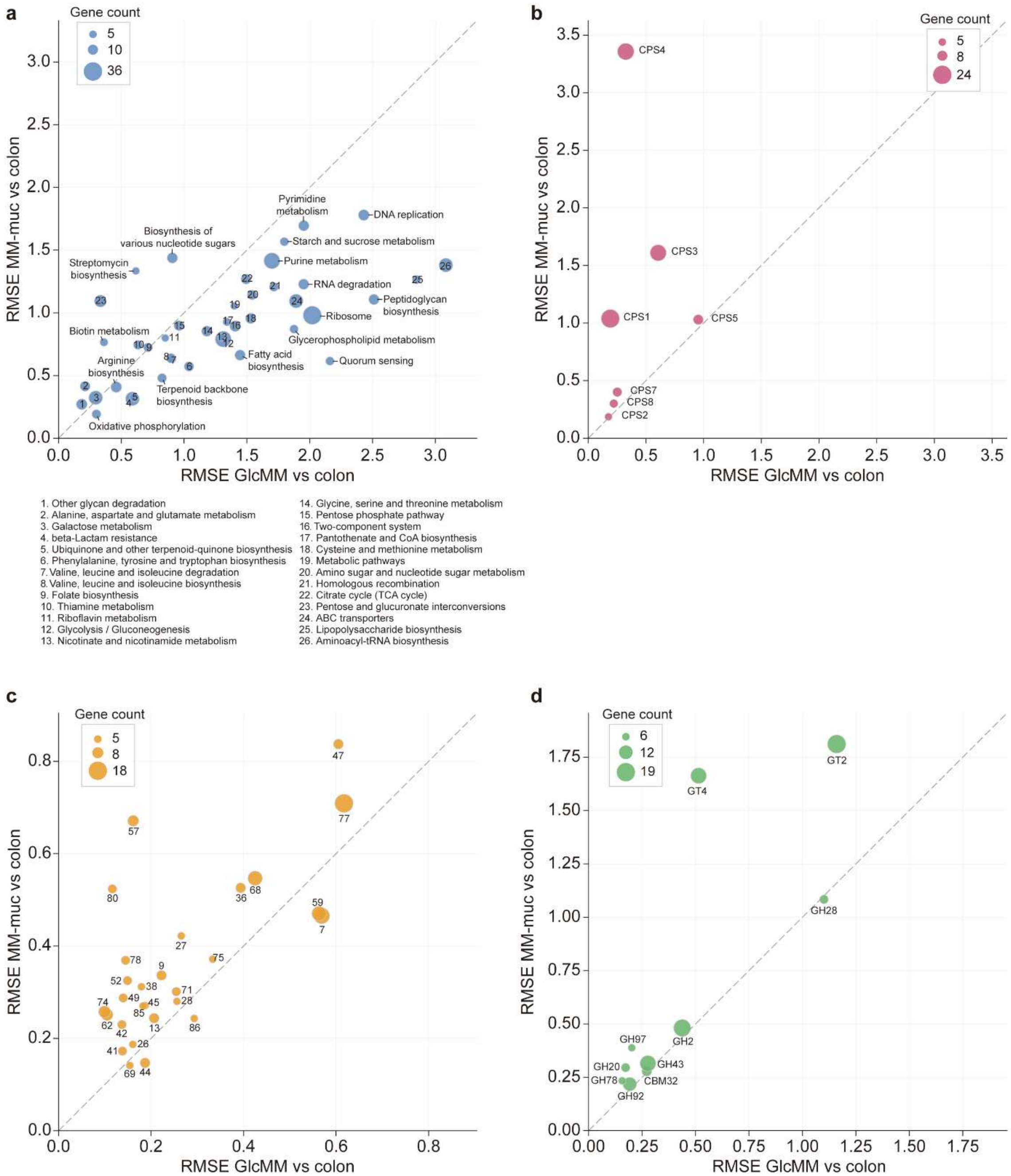


**Figure S15. Category-level root mean square error (RMSE) comparison of in vitro fitness profiles against the in vivo colon profile. (a)** KEGG categories. **(b)** CPS loci. **(c)** PULs. **(d)** CAZy families. For each functional subcategory, the RMSE between MM-muc and colon z-scores is plotted on the y axis, and the RMSE between GlcMM and colon z-scores is plotted on the x axis. Each point represents one subcategory, and circle size indicates the number of genes assigned to that subcategory. The dashed diagonal denotes equal RMSE between the two *in vitro* conditions.

## SUPPLEMENTAL REFERENCE


1. E. A. Cameron, K. J. Kwiatkowski, B. H. Lee, et al., "Multifunctional Nutrient-Binding Proteins Adapt Human Symbiotic Bacteria for Glycan Competition in the Gut by Separately Promoting Enhanced Sensing and Catalysis," *mBio* 5 (2014): e01441–14.
2. Y. Narimatsu, C. Pleguezuelos-Manzano, D. Hornikx, et al., "Discovery of a Secreted *Bacteroides Fragilis* Mucinase That Cleaves Mucins with Bis-T O-Glycans through a Carbohydrate Binding Module-Dependent Mechanism," *Gut Microbes* 18 (2026): 2644983.
3. D. A. Ndeh, S. Nakjang, K. J. Kwiatkowski, et al., "A *Bacteroides thetaiotaomicron* Genetic Locus Encodes Activities Consistent with Mucin *O*-Glycoprotein Processing and N-Acetylgalactosamine Metabolism," *Nature Communications* 16 (2025): 3485.
4. E. C. Martens, R. Roth, J. E. Heuser, and J. I. Gordon, "Coordinate Regulation of Glycan Degradation and Polysaccharide Capsule Biosynthesis by a Prominent Human Gut Symbiont," *Journal of Biological Chemistry* 284 (2009): 18445–57.
5. E. C. Martens, H. C. Chiang, and J. I. Gordon, "Mucosal Glycan Foraging Enhances Fitness and Transmission of a Saccharolytic Human Gut Bacterial Symbiont," *Cell Host & Microbe* 4 (2008): 447–57.
6. T. E. Rogers, N. A. Pudlo, N. M. Koropatkin, et al., "Dynamic Responses of *Bacteroides thetaiotaomicron* during Growth on Glycan Mixtures," *Molecular Microbiology* 88 (2013): 876–90.
7. S. Nakjang, D. A. Ndeh, A. Wipat, et al., "A Novel Extracellular Metallopeptidase Domain Shared by Animal Host-Associated Mutualistic and Pathogenic Microbes," *PloS One* 7 (2012): e30287.
8. A. Benjdia, E. C. Martens, J. I. Gordon, and O. Berteau, "Sulfatases and a Radical S-Adenosyl-L-Methionine (Adomet) Enzyme Are Key for Mucosal Foraging and Fitness of the Prominent Human Gut Symbiont, *Bacteroides thetaiotaomicron*," *Journal of Biological Chemistry* 286 (2011): 25973–82.
9. A. P. Gunning, A. R. Kirby, C. Fuell, et al., "Mining the "Glycocode"--Exploring the Spatial Distribution of Glycans in Gastrointestinal Mucin Using Force Spectroscopy," *FASEB Journal* 27 (2013): 2342–54.
10. D. A. Ravcheev and I. Thiele, "Comparative Genomic Analysis of the Human Gut Microbiome Reveals a Broad Distribution of Metabolic Pathways for the Degradation of Host-Synthetized Mucin Glycans and Utilization of Mucin-Derived Monosaccharides," *Frontiers in Genetics* 8 (2017): 111.
11. L. V. Hooper, J. Xu, P. G. Falk, et al., "A Molecular Sensor That Allows a Gut Commensal to Control Its Nutrient Foundation in a Competitive Ecosystem," *Proceedings of the National Academy of Sciences of the United States of America* 96 (1999): 9833–8.
12. P. C. Kashyap, A. Marcobal, L. K. Ursell, et al., "Genetically Dictated Change in Host Mucus Carbohydrate Landscape Exerts a Diet-Dependent Effect on the Gut Microbiota," *Proceedings of the National Academy of Sciences of the United States of America* 110 (2013): 17059–64.
13. J. Xu, M. K. Bjursell, J. Himrod, et al., "A Genomic View of the Human-*Bacteroides thetaiotaomicron* Symbiosis," *Science* 299 (2003): 2074–6.

14. H. Liu, A. L. Shiver, M. N. Price, et al., "Functional Genetics of Human Gut Commensal *Bacteroides thetaiotaomicron* Reveals Metabolic Requirements for Growth across Environments," *Cell Reports* 34 (2021): 108789.
15. D. Ryan, L. Jenniches, S. Reichardt, et al., "A High-Resolution Transcriptome Map Identifies Small Rna Regulation of Metabolism in the Gut Microbe *Bacteroides thetaiotaomicron*," *Nature Communications* 11 (2020): 3557.
16. A. S. Ruttiger, D. Ryan, L. Spiga, et al., "The Global RNA-Binding Protein Rbpb is a Regulator of Polysaccharide Utilization in *Bacteroides thetaiotaomicron*," *Nature Communications* 16 (2025): 208.
17. K. Kim, D. Choe, S. C. Kim, et al., "Transcriptional Regulatory Networks of the Human Gut Symbiont *Bacteroides thetaiotaomicron* are Uncovered Using Machine Learning," *Nucleic Acids Research* 53 (2025): gkaf1166.
18. A. V. Sastry, Y. Gao, R. Szubin, et al., "The *Escherichia coli* Transcriptome Mostly Consists of Independently Regulated Modules," *Nature Communications* 10 (2019): 5536.
19. K. Kim, E. A. Catoiu, Y. Lee, et al., "The iModulon Framework: How X-Ai Reveals Microbial Regulatory Logic," *mSystems* (2026): e0022826.
